# Discrepancies between self-reported medication in adherence and indirect measurement adherence among patients undergoing antiretroviral therapy: a systematic review

**DOI:** 10.1186/s40249-024-01221-4

**Published:** 2024-07-05

**Authors:** Rujun Liao, Zihuan Tang, Na Zhang, Lin Hu, Zongqi Chang, Jiayi Ren, Xuefei Bai, Jinhong Shi, Sisi Fan, Rong Pei, Liang Du, Tao Zhang

**Affiliations:** 1 Center of Infectious Diseases, Research Center of Clinical Epidemiology and Evidence-Based Medicine, Innovation Insititute for Integration of Medicine and Engineering, West China Hospital, Sichuan University, Chengdu, 610041 Sichuan People’s Republic of China; 2 Present Address: Department of Epidemiology and Health Statistics, West China School of Public Health and West China Fourth Hospital, Sichuan University, Chengdu, 610041 Sichuan People’s Republic of China; 3 Present Address: Sichuan Center for Disease Control and Prevention, Chengdu, 610041 Sichuan People’s Republic of China; 4 School of Public Health, Chengdu University of Traditional Chinese Medicine, Chengdu, 611130 Sichuan People’s Republic of China

**Keywords:** HIV, AIDS, Antiretroviral therapy, Medication adherence, Self-report, Meta-epidemiological one-step analysis, Network meta-analysis

## Abstract

**Background:**

Given the critical importance of medication adherence in HIV/AIDS treatment, this study aims to compare medication adherence measured by self-report (SR) and indirect measurement among antiretroviral therapy (ART) patients, exploring the differences of adherence results measured by different tools.

**Methods:**

We systematically searched PubMed, Embase, and the Cochrane Library to identify all relevant literature published up to November 22, 2023, without language restrictions, reporting adherence to ART measured by both SR and indirect measurement methods, while also analyzing individual and group adherence separately. Discrepancies between SR and indirect measurement results were assessed using the Mann–Whitney U test or Wilcoxon signed-rank test, with correlations evaluated using the Pearson correlation coefficient. Following one-to-one comparisons, meta-epidemiological one-step analysis was conducted, and network meta-analysis techniques were applied to compare results obtained through specific adherence assessment tools reported in the identified articles.

**Results:**

The analysis encompassed 65 original studies involving 13,667 HIV/AIDS patients, leading to 112 one-to-one comparisons between SR and indirect measurement tools. Statistically significant differences were observed between SR and indirect measurement tools regarding both individual and group adherence (*P* < 0.05), with Pearson correlation coefficients of 0.843 for individual adherence and 0.684 for group adherence. During meta-epidemiological one-step analysis, SR-measured adherence was determined to be 3.94% (95% *CI*: -4.48–13.44%) higher for individual adherence and 16.14% (95% *CI*: 0.81–18.84%) higher for group adherence compared to indirectly measured results. Subgroup analysis indicated that factors such as the year of reporting and geographic region appeared to influence the discrepancies between SR and indirect measurements. Furthermore, network meta-analysis revealed that for both individual and group adherence, the results obtained from most SR and indirect measurement tools were higher than those from electronic monitoring devices, with some demonstrating statistical significance (*P* < 0.05).

**Conclusions:**

The findings underscored the complexity of accurately measuring medication adherence among ART patients. Significant variability was observed across studies, with self-report methods showing a significant tendency towards overestimation. Year of reporting, geographic region, and adherence measurement tools appeared to influence the differences between SR and indirect measurements. Future research should focus on developing and validating integrated adherence measurements that can combine SR data with indirect measures to achieve a more comprehensive understanding of adherence behaviors.

**Graphical Abstract:**

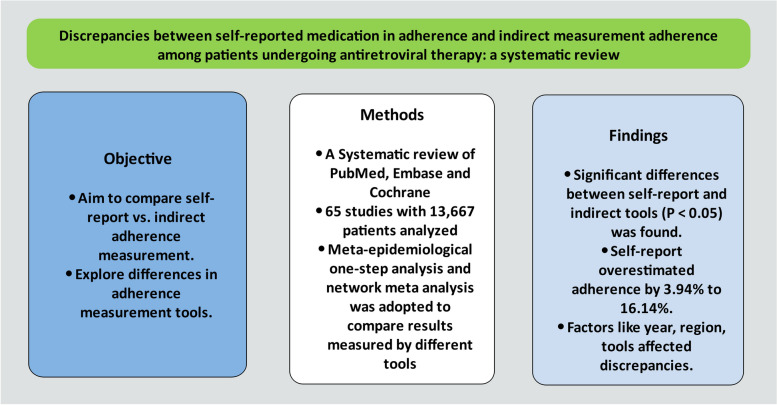

**Supplementary Information:**

The online version contains supplementary material available at 10.1186/s40249-024-01221-4.

## Background

According to the latest report from the Joint United Nations Programme on HIV/AIDS (UNAIDS), as of the end of 2022, there were 1.3 million new HIV infections and 39 million people living with HIV patients globally [1]. The ongoing burden of HIV/AIDS remains significant. Antiretroviral therapy (ART) stands as a cornerstone in HIV/AIDS treatment, with medication adherence playing a pivotal role in its success. HIV/AIDS patients are required to achieve a medication adherence rate of at least 95% for optimal therapeutic outcomes [2, 3]. However, managing a chronic disease like HIV/AIDS presents various challenges for patients, including coping with medication side effects, adjusting to lifestyle changes, and enduring long-term treatment pressures, all of which can contribute to reduced medication adherence. Consequently, monitoring and assessing medication adherence among HIV/AIDS patients is imperative.

Presently, self-report stands as a frequently utilized method for measuring medication adherence, yet its reliability is subject to scrutiny owing to potential biases like recall bias and reporting bias, often resulting in overestimations [4]. Assessments can offer valuable and actionable insights, particularly in settings prioritizing speed, efficiency, and cost-effectiveness, notably in resource-constrained areas. Meanwhile, indirect measurement tools, such as pharmacy refill rates, electronic monitoring devices (EMD), and biomarker analysis, have gained increasing recognition for their potential to provide objective and quantifiable data on patient adherence behaviors. These tools hold particular significance in ART, where precise adherence monitoring is imperative for achieving viral suppression and averting resistance development. However, the use of indirect measurement methods often entails additional equipment and personnel, restricting their utility in resource-limited settings. Consequently, building upon prior research comparing self-report and indirect adherence measures, this study employs systematic review and meta-analysis techniques to explore disparities, correlations, and potential influencing factors between self-report and indirect measures. Through this analysis, the study aims to shed light on the reliability and accuracy of various measurement tools.

## Methods

### Search strategy

We conducted a comprehensive search across PubMed, Embase, and the Cochrane Library to identify all relevant literature published up to November 22, 2023, without language restrictions. Our search utilized a combination of disease and treatment terms ('HIV/AIDS', 'antiretroviral therapy', etc.), measurement indices ('medication adherence', 'patient compliance', etc.), and measurement tools ('self-report', 'pill count', 'medical record', etc.), with the three components linked by 'AND'. Further details of the search strategy can be found in Supplementary Material 1. This systematic review has been registered with INPLASY, and the record is publicly available on inplasy.com. Our registration number is INPLASY2023110040, and DOI number is 10.37766/inplasy2023.11.0040.

### Eligibility criteria

The studies included in our research adhered to the following criteria: (1) observational or interventional study design, (2) focusing on HIV ART treatment, (3) employing both self-report and indirect measurement methods to assess medication adherence, and (4) possessing complete and accessible data.

Studies meeting the following criteria were excluded: (1) non-English literature, (2) duplicate publications or redundant data, (3) case reports or review literature, (4) studies lacking accessible or incomplete data, (5) comparative studies of different drugs for the same disease, to mitigate selection reporting bias, (6) studies involving patients with comorbidities potentially affecting medication adherence, such as psychosis and pregnancy status. Furthermore, for studies implementing adherence intervention measures, only data from blank control groups or baseline assessments were included.

### Study selection

Based on the title, abstract, and keywords, literature preliminarily meeting the requirements will be selected. Subsequently, through a thorough examination of the full text, literature meeting the inclusion criteria will be determined.

### Data extraction

The following information will be extracted from the selected literature: (1) basic information: title, author, publication time, study type, research subject, sample size, etc., (2) self-report medication adherence data: measurement tools, measurement time point, results of measurement, etc., (3) indirect measurement medication adherence data: measurement tools, measurement time point, results of measurement, etc., (4) additional information such as research purpose, experimental design, and quality control measures.

Two senior experts independently screen the abstracts and full texts of the articles. In case of discordant views among the experts, they engage in discussions to reach a consensus, or if needed, a third expert is invited to arbitrate. Adherence data extracted from literature are categorized into two groups: individual adherence, denoting the average adherence rates across all individuals; and group adherence, representing the percentage of individuals in the group meeting the specified adherence criteria (e.g., 95%) as outlined in the article.

### Quality evaluation

A checklist recommended by the Agency for Healthcare Research and Quality (AHRQ) was used for assess the quality of included research, with only data from cross-sectional studies and baseline or blank control groups of controlled design research being analyzed. This scale comprised 11 items and evaluated five common risks of bias: literature selection bias, performance bias, follow-up bias, measurement bias, and reporting bias (see Supplementary Material 2). Each fulfilled item was awarded a point. Articles scoring 8 or above were deemed to be of high methodological quality, while those scoring below 8 were excluded from our study.

### Meta-analysis

Individual and group adherence were analyzed separately. The extracted data was subjected to a random effects model or fixed effects model according to the heterogeneity test results to calculate the standardized mean difference (*SMD*), and calculate a 95% confidence interval (*CI*). Statistical differences between the two reporting methods were assessed using the Mann–Whitney U test or Wilcoxon sign-rank test, while correlations were measured using the Pearson correlation coefficient. Organizing original studies into one-to-one comparisons between self-report and indirect measurement tools, a one-step meta-epidemiological method was utilized to analyze the degree of exaggeration or underestimation of adherence results compared with indirect measurement using SR. Depending on heterogeneity, either the random effect model or fixed effect model was selected for statistical analysis, with restricted maximum likelihood estimation (REML) adopted as the regression method.

The central concept of meta-epidemiological one-step analysis involves linking adherence results and adherence measurement tools from each independent original study. Adherence results serve as the dependent variable, while adherence measurement tools act as the independent variable in regression analysis. The resulting regression coefficient is converted into the original value, representing the extent of exaggeration or underestimation of adherence results compared to both SR and indirect measurement methods. For group adherence results, the degree of exaggeration or underestimation is represented by the risk ratio (RR) [5], and the extent of exaggeration or underestimation of individual adherence results is quantified by the SMD value. If RR > 1 or SMD > 1, it indicates that SR may exaggerate adherence results compared to indirect measurement, while the opposite is true if RR < 1 or SMD < 1. Sub-group analysis was conducted to explore significant heterogeneity, and network meta-analysis (NMA) was employed to simultaneously compare adherence results measured by multiple tools, integrating both direct and indirect comparisons across studies. Publication bias was assessed using funnel plots and confirmed with Egger’s test. In cases of significant bias, sensitivity analysis methods were employed to evaluate the stability and reliability of the network meta-analysis results. All statistical analyses were performed using Stata software (Version 15, Stata Corporation, College Station, TX, USA) and R (version 4.2.1, R foundation for Statistical Computing, Vienna, Austria).

## Results

### Characteristics of researches included

**Fig. 1:**
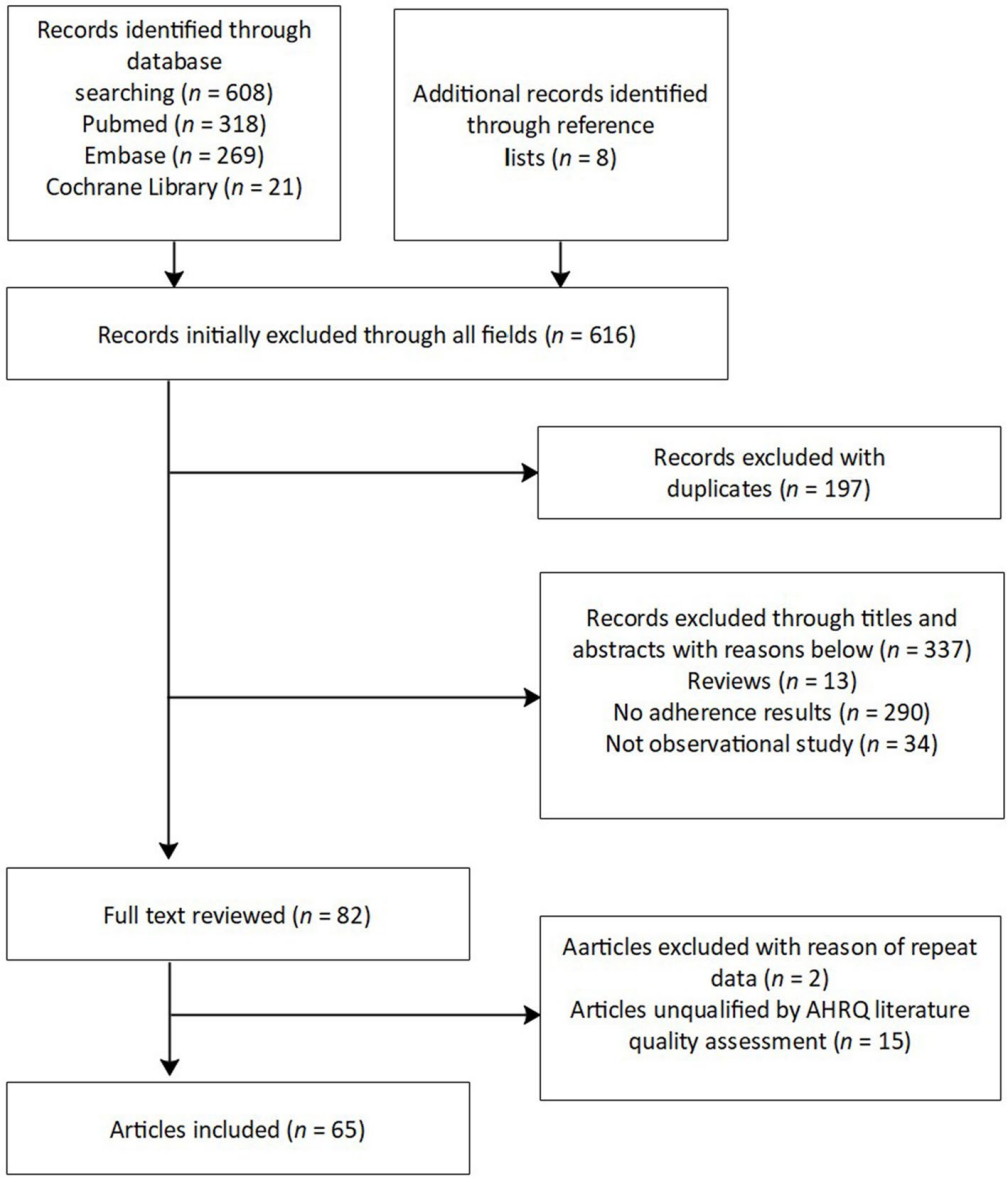
Flow diagram for study screening and selection process. *AHRQ* Agency for Healthcare Research and Quality Evaluation Form. AHRQ agency for healthcare research and quality

Out of the 608 articles retrieved, 65 studies with a total number of 13,667 patients were included in the meta-analysis (Fig. 1). These studies spanned publication years from 2006 to 2022 and originated from various geographical regions including North America, Africa, South America, and Asia. In terms of study design, 56 studies were cohort studies, 5 were cross-sectional studies, and 4 were randomized controlled trials (RCTs). Data from cohort studies and RCTs were limited to baseline or blank control groups.

Given that only non-intervention results were considered, the AHRQ report assessment was utilized for quality evaluation. Following the AHRQ literature quality assessment, 65 articles classified as adherent were included for meta-analysis, with 59 of these articles reporting comparisons of measurement results from multiple medication adherence measurement tools.

After organizing one-to-one comparisons between self-report and indirect measurement tools, a total of 112 comparisons were made, with 30 comparisons on individual adherence rates and 82 comparisons on group adherence rates. One study [6] reported both individual and group adherence.

The SR tools used in the included studies encompassed scales, self-designed questionnaires, qualitative single-item measures, and daily reporting (Table 1A). The scales involved in the analysis were as follows: Morisky, visual analog scale (VAS), AIDS Clinical Trials Group (ACTG), Morisky medication adherence scale-8 Items (MMAS-8), Medication adherence training instrument (MATI), Patient Medication Adherence Questionnaire (PMAQ). In the included studies, the questionnaires uniformly inquire with questions "How many times have you missed your medication in a past period?" and similar queries, allowing for mutual comparison. The indirect measurement tools comprise pill count (PC), pharmacy refill (PR), electronic monitoring device (EMD), biological marker (Bio), and appointment record (APM) (Table 1B). Both Bio and EMD are considered relatively accurate measurement method [7], yet fewer studies involved Bio. Consequently, EMD will be selected as the reference group for subsequent analysis.

**Table 1:** Tools for measuring ART adherence included in the studies

A. SR adherence measurement					
Tools	No. of studies	Time of recall (days)	No. of items	Cronbach's α	Accessibility
Morisky	1	7–30	4	0.61–0.83	Free
VAS	15	3–30	1	—^*^	Free
ACTG	9	4	4–6	0.70–0.90	Free
MMAS-8	2	7–30	8	0.68–0.83	Charge
MATI	1	30	1	—^*^	Free
PMAQ	1	NR^*^	NR^*^	NR^*^	Free
Questionnaire	48	2–180	1–58	—^*^	Free
B. Indirect adherence measurement					
Tools		No. of studies	Time of detective (days)	Specifc device	Charge
PC		40	3–180	No	Low
PR		14	28–90	No	Low
EMD		22	14–90	Yes	High
Bio		5	—^*^	Yes	High
APM		1	NR^*^	No	Low

^*^NR Not reported; “—” stands for not applicable. *VAS* Visual analog scale, *ACTG* Visual analog scale, *MMAS-8* Visual analog scale, *MATI* Medication adherence training instrument, *PMAQ* Patient medication adherence questionnaire, *PC* Pill counting, *PR* Pharmacy refill, *EMD* Electronic monitoring devices, *Bio* Biological maker, *APM* Appointment record

In our analysis, individual adherence is depicted as the adherence rate, defined as the percentage of patients' medication possession rate (doses of prescribed medicine taken/prescribed doses). In the measurement results of the electronic monitoring device (EMD), both dose adherence and timing adherence were reported. However, for comparability with results measured by other tools, only the dose adherence results are included. For group adherence, the percentage of adhered patients determined based on SR tools and indirect measurement results is reported. Among the total of 40 articles reporting adherence rate thresholds for determining adherence in patients, 74.1% of them were set at 95% (40 out of 54). In articles published after 2016, except for two [8, 9], the threshold remained consistently set at 95%. Since both SR and indirect measurement tools utilize consistent criteria for determining adherence, literature with different adherence rate thresholds can be compared.

### Differences between self-report and indirect measurement tools in measuring individual adherence

There are 17 peer reviewed articles [6, 10–25] reported SR adherence and adherence results measured by indirect tools simultaneously, resulting in a total of 30 comparisons. Egger test did not find the asymmetry on the funnel plot (*P* = 0.1437) (Supplementary Fig. 1).

### Comparison analysis of individual adherence results by self-report and indirect measurement

Based on the results of the Shapiro–Wilk normality test, the mean distributions of SR results and indirectly measured results do not conform to a normal distribution (*P* < 0.001). Therefore, a non-parametric test, the Mann–Whitney U test, was selected to compare the two sets of data. The analysis revealed a significant difference between SR adherence and adherence measured indirectly (*U* = 596.00, *P* = 0.007). This suggests that the adherence results obtained from the two measurement methods significantly differ statistically, with a notable numerical variance between SR adherence and adherence measured by other tools.

The Pearson correlation coefficient between SR adherence results and indirectly measured adherence results is 0.843 (*P* < 0.0001), indicating a positive linear relationship. This finding suggests that despite differences in adherence results from SR and indirect measurement, there exists a considerable level of coherence between them.

### Meta-epidemiological one-step analysis of comparison of self-report and indirect measurement tools

A total of 30 individual adherence rate comparisons, focusing on the percentage of medication taken, were examined. The comparison findings between SR adherence and indirectly measured adherence are detailed in Table 2. Employing a meta-epidemiological one-step analysis, SR medication adherence results were compared with those obtained through indirect measurements, with the latter serving as the control and REML utilized as the regression method. Among the 30 comparison analyzed, SR medication adherence was observed to be 3.94% higher than adherence measured indirectly, with a standard error of 0.045 (95% *CI*: -4.48%–13.44%, *P* = 0.380), suggesting no statistically significant difference.

**Table 2:** Sub-group analysis of differences in the results of different measurement tools for evaluating individual adherence of HIV patients

	Number of objectives	Exp (b)^*^	95% *CI*	*SE*	*t*	*P*	*I*^*2*^
Total	60	1.0394	0.9524–1.1344	0.0454	0.88	0.380	99.60%
Region							
North America	26	1.0561	0.8598–1.2591	0.0894	0.65	0.525	99.75%
Africa	26	1.0281	0.9499–1.1127	0.0394	0.72	0.477	99.82%
Asia	4	1.0310	0.7523–1.4130	0.0755	0.42	0.717	71.85%
Report year							
Before 2016	52	1.0424	0.9449–1.1498	0.0509	0.85	0.400	99.75%
After 2016	8	1.0212	0.8205–1.2710	0.0913	0.23	0.822	98.89%

^*^One comparison in South America and one in Europe were not included in this analysis. *Exp(b)* represents for the coefficient for each *SMD* change, *CI* Confidence interval, *SE* Standard error

### Sub-group analysis

Subgroup analysis by region revealed that in all three regions included in the study, adherence rates determined by SR were higher compared to indirect measurements. However, the difference was not statistically significant (*P* > 0.05). Additionally, no significant difference was found in the degree of SR overestimation between developed regions and resource-limited areas.

The subgroup analysis regarding the reporting time indicated a decrease in the degree of SR overestimation in literature published in 2016 and thereafter. However, there was no significant difference observed between them (Table 2).

### Network meta-analysis of different measurement tools

For studies reporting individual adherence, the network meta-analysis (NMA) was conducted, incorporating data from 17 studies, which involved 37 pairwise comparisons across six distinct adherence measurement methods.

We compared the measurement results of EMD against those of 11 other adherence measurement tools across 37 comparisons, calculating the SMD through a random effects model (*I*^*2*^ = 100%). The analysis revealed that, with the exception of questionnaire with a recall period of 30 days (Q30), the majority of adherence measurement tools reported significantly higher adherence outcomes(all *P* < 0.001) compared to EMD, such as ACTG (SMD = 1.0532), PC (SMD = 1.0043), and (SMD = 1.4769), indicating a potential for these tools to overestimate adherence levels. Notably, Q30's outcomes were significantly lower than EMD, suggesting a potential for inaccuracy (Table 3).

**Table 3:** Differences in the results of different measurement tools for evaluating individual adherence of HIV patients through a network meta-analysis (EMD as reference)

Tool	SMD	95% *CI*	*z*	*P*
ACTG	1.0532	0.7974–1.3090	8.07	< 0.0001
EMD				
PC	1.0043	0.8280–1.1806	11.16	< 0.0001
PR	0.8943	0.3371–1.4515	3.15	0.0017
Q2-4^*^	1.4769	1.2010–1.7527	10.49	< 0.0001
Q30^*^	-0.6684	–0.9602–0.3766	-4.49	< 0.0001
Q7^*^	1.1111	0.8484–1.3738	8.29	< 0.0001
Qi^*^	0.6334	0.3776–0.8891	4.85	< 0.0001
VAS3^*^	1.0740	0.7695–1.3786	6.91	< 0.0001
VAS30^*^	0.9432	0.7656–1.1207	10.41	< 0.0001
VAS7^*^	1.4527	0.9272–1.9781	5.42	< 0.0001

^*^ACTG AIDS Clinical Trials Group Adherence Questionnaire, *EMD* Electronic monitoring devices, *PC* Pill counting, *PR* Pharmacy refill, *Q* Qustionare, *VAS* Visual Analog Scale. The numbers following the measurement tools represent the number of days in the recall period for the scale, "i" stands for single-item qualitative measurement

**Fig. 2:**
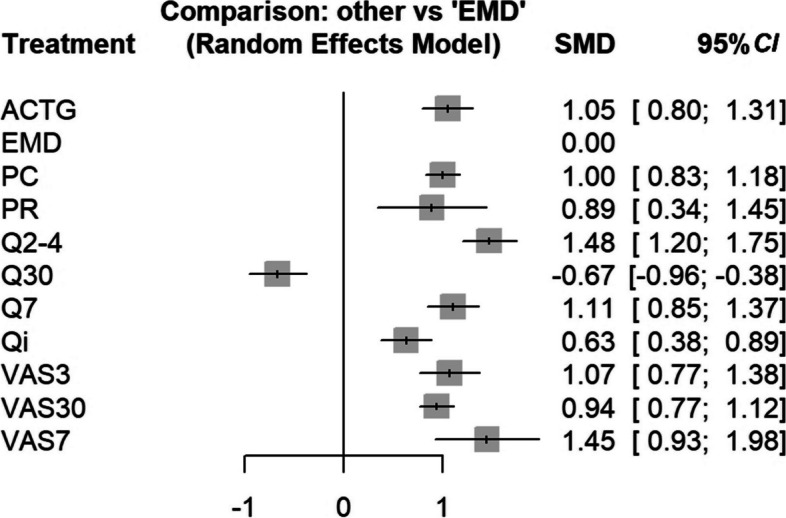
Forest plot of differences in the results of different measurement tools for evaluating individual adherence of HIV patients through a network meta-analysis. *ACTG* AIDS Clinical Trials Group Adherence Questionnaire, *EMD* electronic monitoring devices, *PC* pill counting, *PR* pharmacy refill, *Q* qustionare, *VAS* Visual Analog Scale. The numbers following the measurement tools represent the number of days in the recall period for the scale, "i" stands for single-item qualitative measurement

Examining the forest plot (Fig. 2), it becomes apparent that the results of Q30 and single-item qualitative measurement (Qi) are the closest to those of EMD.

Sensitivity analysis found that the study with the largest effect size was the comparison of PC and Q30 in the study by Wall et al. [25], and the sample size of the study by Haberer et al. [18] was too small (*n* < 20). Even after excluding the study with the largest effect size, still statistical significance persisted for most tools compare to EMD (*P* < 0.05), except for PR, Q30 and Qi (Supplementary Table 1A). Furthermore, following the exclusion of the study with a small sample size, all tools continued to exhibit significant differences compared to EMD. This suggests that despite the removal of studies with either the largest effect size or small sample sizes, the significant effects of the tools relative to EMD remain robust. Although heterogeneity was reduced, it remained high, indicating the robustness of the network meta-analysis conclusions to a certain extent.

Moreover, this study received scores above 8 from AHRQ, indicating high quality, and was not exclude in the overall study therefore.

The Begg’s test of funnel plot asymmetry found no significant relationship between effect size and its precision, indicating no evidence of publication bias (*t* = 0.06, *df* = 35, *P* = 0.9494).

### Differences between self-report and indirect measurement tools in measuring group adherence

Forty-nine peer-reviewed articles [6, 8, 9, 26–71] provided data on adherent ratios measured simultaneously by SR and indirect tools, totaling 82 comparisons reported the adherent ratios measured by SR and indirect tools at the same time, with totally 82 comparisons. Egger test confirmed asymmetry in the funnel plot (*P* < 0.0001) (Supplementary Fig. 2). Even after excluding studies with the largest effect sizes, the results of sensitivity analyses remained significant (Supplementary Table 2).

### Comparison analysis of group adherence results by self-report and indirect measurement

Based on the results of the Shapiro–Wilk normality test, it was found that the mean distributions of SR results and indirectly measured results do not adhere to a normal distribution (*P* < 0.01). Consequently, a non-parametric test was selected. The Wilcoxon signed-rank test revealed that there is a statistically significant difference in adherence patient percentages obtained by the two measurement tools (W = 327.00, *P* < 0.0001). This indicates a significant disparity in the proportion of adherent patients as determined by the results of the two adherence measurement methods.

Moreover, the Pearson correlation coefficient between SR adherence outcomes and indirectly measured adherence outcomes was calculated to be 0.684 (*P* < 0.0001), suggesting indicating a moderate to strong positive correlation. This finding implies that despite variations in adherent proportions measured by the two methods, they exhibit a degree of consistency, both reflecting patients' medication-taking behavior.

### Meta-epidemiological one-step analysis of comparison of self-report and indirect measurement tools

A meta-epidemiological one-step analysis, comparing proportions of adherent patients classified based on SR with results based on indirect measurement, utilized indirect measurement as the control and employed REML as the regression method. This analysis encompassed 82 comparisons. The proportion of patients classified as adherent through self-report was found to be 16.14% higher than that measured indirectly, with a standard error of 0.038 (95% *CI*: 4.81–18.84%, *P* = 0.001), indicating a statistically significant difference.

### Sub-group analysis

The regional subgroup study revealed that across all four regions included in the study, adherence proportions measured by SR were consistently higher than those measured indirectly. This difference reached statistical significance in Africa (*P* < 0.001) and South America (*P* < 0.001), and approached significant in Asia (*P* = 0.086). Additionally, in the subgroup analysis based on reporting time, it was found that the adherence proportion determined by SR results reported before 2016 exceeded that measured by indirect methods by 18.18%. Conversely, after 2016, this difference decreased slightly to 13.04%. These findings are summarized in Table 4.

**Table 4:** Sub-group analysis of differences in the results of different measurement tools for evaluating group adherence of HIV patients

	Number of objectives	Exp (b)^*^	95% *CI*	*SE*	*t*	*P*	*I*^*2*^
Total	164	1.1614	1.0884–1.2394	0.0382	4.55	< 0.001	98.51%
Region							
North America	44	1.1426	0.9735–1.3412	0.0907	1.68	0.100	98.36%
Africa	94	1.1318	1.0671–1.2004	0.0335	4.18	< 0.001	99.96%
South America	14	1.4329	1.2324–1.6659	0.1007	5.12	< 0.001	98.57%
Asia	14	1.1680	0.9747–1.3996	0.0970	1.87	0.086	99.79%
Report year							
Before 2016	104	1.1818	1.2354–1.5725	0.0568	3.47	< 0.001	98.96%
2016 and after	64	1.1304	1.0508–1.2161	0.0527	3.35	0.001	98.52%

^*^Exp(b) represents the coefficient for each *RR* change. *CI* Confidence interval, *SE* Standard error

### Network meta-analysis of the different measuring tools

**Fig. 3:**
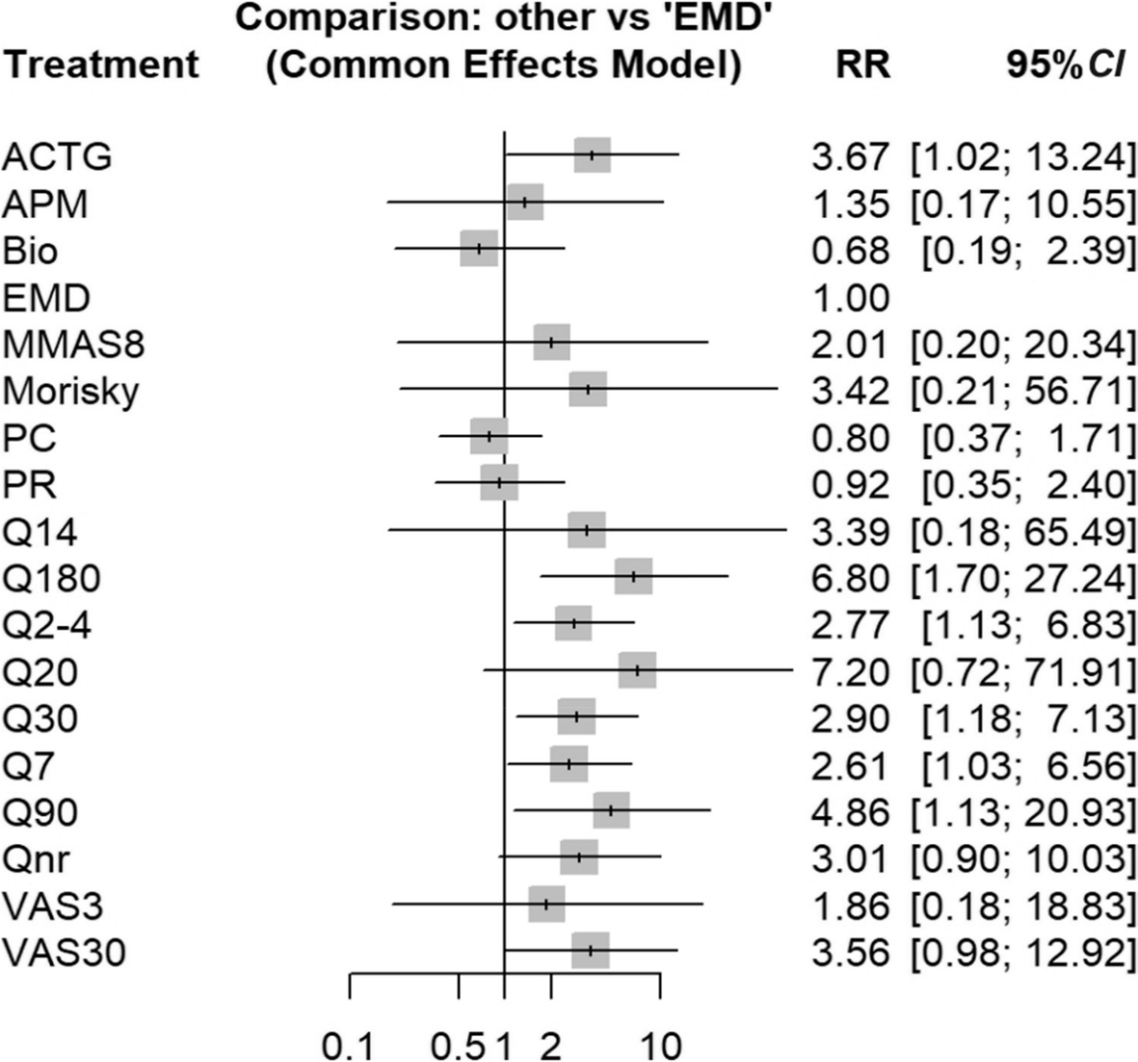
Forest plot of differences in the results of different measurement tools for evaluating group adherence of HIV patients through a network meta-analysis. *ACTG* AIDS Clinical Trials Group Adherence Questionnaire, *APM* appointment record, *Bio* biological maker, *EMD* electronic monitoring devices, *MMAS-8* Morisky Medication Adherence Scale-8 Items, *PC* pill counting, *PR* pharmacy refill, *Q* qustionare, *VAS* Visual Analog Scale. The numbers following the measurement tools represent the number of days in the recall period for the scale. "i" stands for single-item qualitative measurement, and "nr" stands for not reported recall period

In our network meta-analysis, we scrutinized 10 distinct t tools for measuring adherence to ART, encompassing 116 pairwise comparisons (Fig. 3). The results of heterogeneity and consistency tests revealed an *I*^2^ < 50%, and tests for consistency within and between designs also indicated no significant inconsistency. Consequently, a common effect model was selected for further analysis. Previous research suggests that the EMD provide a more accurate measure of patient adherence compared to other methods [72, 73], therefore, EMD results are considered as a reference. Under the common effect model, certain adherence measurement tools, including ACTG, Q180, Q2-4, Q30, Q7, and Q90, demonstrated a proportionally higher number of adherent patients compared to EMD, with the differences statistically significant (all *P* < 0.05) (Table 5).

**Table 5:** Differences in the results of different measurement tools for evaluating group adherence of HIV patients through a network meta-analysis (EMD as reference)

Tool	RR	95% *CI*	z	*P*
ACTG	3.6672	1.0159–13.2376	1.98	0.0472
APM	1.3498	0.1726–10.5550	0.29	0.7750
Bio	0.6809	0.1937–2.3934	-0.60	0.5490
EMD				
MMAS-8	2.0060	0.1979–20.3378	0.59	0.5558
Morisky	3.4212	0.2064–56.7080	0.86	0.3906
PC	0.7955	0.3694–1.7131	-0.58	0.5588
PR	0.9219	0.3538–2.4025	-0.17	0.8679
Q14^*^	3.3898	0.1755–65.4903	0.81	0.4191
Q180^*^	6.7978	1.6963–27.2420	2.71	0.0068
Q2-4^*^	2.7717	1.1254–6.8264	2.22	0.0266
Q20^*^	7.2029	0.7215–71.9093	1.68	0.0926
Q308	2.9012	1.1813–7.1255	2.32	0.0202
Q78	2.6056	1.0349–6.5601	2.03	0.0421
Q90^*^	4.8622	1.1294–20.9334	2.12	0.0337
Qnr^*^	3.0132	0.9048–10.0349	1.80	0.0724
VAS3^*^	1.8604	0.1838–18.8287	0.53	0.5991
VAS30^*^	3.5566	0.9790–12.9200	1.93	0.0539

^*^ACTG AIDS Clinical Trials Group Adherence Questionnaire, *APM* Appointment record, *Bio* Biological maker, *EMD* Electronic monitoring devices, *MMAS-8* Morisky Medication Adherence Scale-8 Items, *PC* Pill counting, *PR* Pharmacy refill, *Q* Qustionare, *VAS* Visual Analog Scale. The numbers following the measurement tools represent the number of days in the recall period for the scale. "i" stands for single-item qualitative measurement, and "nr" stands for not reported recall period

Sensitivity analysis identified the largest effect size study by Vaz et al. [64] focusing on the comparison between PC and questionnaires with a recall period of 4 days. Additionally, and the sample size of the studies by Da costa et al. [53] and Wiens et al. [48] was too small (*n* < 20). After excluding the study with the largest effect size or small sample size, methods such as ACTG and questionnaires continued to demonstrate significantly higher adherence results compared to EMD, aligning with previous analysis findings (Supplementary Table 2). This indicates a degree of robustness in the results of the network meta-analysis to the exclusion of individual studies. These studies received an 11 score from AHRQ, indicating high quality, and therefore retained in the analysis.

The Begg’s test of funnel plot asymmetry found no significant relationship between effect size and its precision, indicating no evidence of publication bias (*t* = 1.21, *df* = 114, *P* = 0.2301).

## Discussion

This study provides a systematic review of discrepancies between SR medication adherence and indirect measurement adherence among HIV/AIDS patients undergoing ART. The findings indicate that SR methods tend to overestimate adherence compared to electronic medication dispensers (EMD) and other indirect measures, corroborating recent research [47].

Key findings reveal that SR adherence tends to be, on average, 3.94% higher than adherence measured indirectly, with a 16.14% higher proportion of patients reported as adherent through self-reports. Egger's test and sensitivity analysis suggest that publication bias does not significantly influence the results.

In terms of group adherence, the statistically significant discrepancy underscores the potential for self-report measures to overestimate adherence [74, 75]. Although individual adherence differences may not always reach statistical significance, the practical significance of a 3.94% overestimation remains noteworthy.

Considering the World Health Organization (WHO)'s "90–90-90" target for 2030, such discrepancies could potentially impact the determination of whether a substantial number of regions meet the criteria.

In the meta-epidemiological one-step analysis of individual adherence, there were no statistically significant differences found among regions or report years. However, for group adherence, subgroup analyses revealed statistically significant differences or ones close to significance between SR and indirect measurement results in South America, Africa, and Asia. This suggests that the degree of overestimation of SR may be influenced by the level of regional development.

Additionally, there was an observed increase in both SR and indirectly measured group adherence rates post-2016 compared to pre-2016. This trend aligns with the global push for achieving the '90–90-90' targets set by UNAIDS in 2014 and subsequently endorsed by the WHO in 2016 [76]. The observed improvement in adherence rates post-2016 suggests that these global initiatives may have had a positive impact on enhancing ART adherence among patients.

The meta-epidemiological one-step analysis conducted in our study provides valuable insights into the factors influencing medication adherence measurement discrepancies among patients undergoing ART. The significant effects identified regarding report time, the utilization of biological markers, pill counting, electronic medication monitoring devices, and the specific study location highlight the complexity of accurately assessing ART adherence.

A novel contribution of this research is the findings from a network meta-analysis, which highlight the variability in patient adherence measurements across different tools. Even in instances where statistical significance was not reached, individual adherence results measured by questionnaire surveys were consistently higher compared to indirectly measured results, indicating potential inaccuracies. SR adherence assessed through questionnaires is susceptible to numerous biases, resulting in an overestimation of adherence [77, 78]. Patients are particularly prone to recall errors, especially as the time interval between drug consumption and assessment increases. They may recall their routine or intention to take medication rather than their actual behavior.

Furthermore, the provision of antiretroviral drugs free of charge, coupled with the global push for achieving the '90–90-90' targets set by WHO, has led to strengthened drug adherence monitoring and supervision in various regions worldwide. Consequently, pharmacy supplementation records may reflect higher medication-taking percentages than the actual situation.

Moreover, our analysis revealed the presence of high heterogeneity in studies comparing adherence measurement tools. This might reflect the diversity in study contexts, including differences in study designs, participant characteristics, and variations in definitions and measurements of adherence. Such high heterogeneity underscores the need for careful consideration of the impact of study design and measurement method selection in adherence research.

Notably, while most of the questionnaire survey presented overestimation compare with EMD, this observation may suggest the need for a calibration model and careful consideration of specific research objectives and participant characteristics when selecting measurement tools in certain scenarios. Since no single measure consistently offers sufficiently high sensitivity or specificity to detect viral non-suppression [79], which serves as the WHO 'gold standard' for confirming treatment response, the calibration model should incorporate the use of biological markers as dependent variables.

In summary, our study provides a comprehensive assessment of the discrepancies within self-report and objective adherence tools, enabling an informed selection of the most appropriate methods for specific research or clinical contexts. By offering a hierarchy of adherence measurement tools, it guides healthcare professionals and researchers in choosing the most effective tools for monitoring and improving patient adherence.

Furthermore, there is a pressing need to explore the integration of digital health technologies in adherence monitoring and estimation. Given the promising approaches offered by various digital technologies, such as electronic healthcare databases, mobile apps, chatbots, and digital pills, integrated tools have the potential not only to enable healthcare providers to track patients’ adherence to prescriptions more accurately but also to facilitate the personalization of treatment regimens and the provision of targeted interventions [80].

## Conclusions

Our systematic review reveals a consistent trend of SR adherence overestimating medication adherence compared to indirect measures among HIV/AIDS patients on antiretroviral therapy. Despite minimal publication bias impact, the discrepancy between SR and indirect measures has practical implications, potentially affecting the evaluation of regional adherence goals, especially in achieving WHO targets. Regional and temporal variations suggest influences of development levels and global initiatives like the '90–90-90' targets. In summary, our study provides crucial insights for selecting appropriate adherence measurement methods, guiding healthcare professionals and researchers, and underscores the potential of digital health technologies for personalized interventions.

### Supplementary Information

Supplementary Material 1.

Supplementary Material 2.

Supplementary Material 3.

Supplementary Material 4.

## Data Availability

All the data was from published journal literature in databases, such as Pubmed, Embase and Cochrane Library.

## Abbreviations

ACTG: AIDS Clinical Trials Group Adherence Questionnaire
AHRQ: Agency for Healthcare Research and Quality
APM: Appointment record
ART: Antiretroviral therapy
Bio: Biological maker
*CI*: Confidence interval
EMD: Electronic monitoring devices
MATI: Medication adherence training instrument
MMAS-8: Morisky Medication Adherence Scale-8 Items
NMA: Network meta-analysis
PC: Pill Counting
PMAQ: Patient Medication Adherence Questionnaire
PR: Pharmacy refill
Q: Questionnaire
Qi: Single-item qualitative measurement
Qnr: Questionnaire not reported recall period
REML: Restricted Maximum Likelihood
RR: Risk ratio
SE: Standard error
SMD: Standardized mean difference
SR: Self report
UNAIDS: Joint United Nations Programme on HIV/AIDS
VAS: Visual Analog Scale
WHO: World Health Organization

## References

[1] UNAIDS. UNAIDS Global AIDS Update 2023: The path that ends AIDS. 2023. Accessed 13 July 2023.

[2] Diabaté S, Alary M, Koffi CK (2007). Determinants of adherence to highly active antiretroviral therapy among HIV-1-infected patients in Côte d'Ivoire. AIDS.

[3] Levy RW, Rayner CR, Fairley CK, Kong DC, Mijch A, Costello K (2004). Multidisciplinary HIV adherence intervention: a randomized study. AIDS Patient Care STDS.

[4] Miller LG, Liu H, Hays RD, Golin CE, Beck CK, Asch SM (2002). How well do clinicians estimate patients' adherence to combination antiretroviral therapy?. J Gen Intern Med.

[5] Li X. Health Statistics. 8th edn: People's Medical Publishing House; 2017.

[6] Nabukeera-Barungi N, Kalyesubula I, Kekitiinwa A, Byakika-Tusiime J, Musoke P (2007). Adherence to antiretroviral therapy in children attending Mulago Hospital. Kampala Ann Trop Paediatr.

[7] Spinelli MA, Haberer JE, Chai PR, Castillo-Mancilla J, Anderson PL, Gandhi M (2020). Approaches to Objectively Measure Antiretroviral Medication Adherence and Drive Adherence Interventions. Curr HIV/AIDS Rep.

[8] Kizindo J, Marealle AI, Mutagonda R, Mlyuka HJ, Mikomangwa WP, Kilonzi M (2022). Adherence to Antiretroviral Therapy Among HIV-Infected Clients Attending Opioid Treatment Program Clinics in Dar es Salaam, Tanzania. Cureus.

[9] Allam RR, Takamiya M, Pant R, Gandham S, Yeldandi VV, Thomas J (2020). Factors associated with non-adherence to antiretroviral therapy among female sex workers living with HIV in Hyderabad. India Int J STD AIDS.

[10] Abiodun O, Ladi-Akinyemi B, Olu-Abiodun O, Sotunsa J, Bamidele F, Adepoju A, et al. A single-blind, parallel design RCT to assess the effectiveness of SMS reminders in improving ART adherence among adolescents living with HIV (STARTA Trial). J Adolesc Health. 2021;68(4):728–36. 10.1016/j.jadohealth.2020.11.016. 10.1016/j.jadohealth.2020.11.016 33342719

[11] Pang Y, Molton JS, Ooi WT, Paton NI, He HG. Preliminary effects of a mobile interactive supervised therapy intervention on people living with HIV: pilot randomized controlled trial. JMIR Mhealth Uhealth. 2020;8(3):e15702. 10.2196/15702. 10.2196/15702 PMC7148554 32217500

[12] Gaifer Z, Boulassel MR. Comparative analysis of two methods of measuring antiretroviral therapy adherence in HIV-infected Omani patients. J Int Assoc Provid AIDS Care. 2019;18:2325958219867316. 10.1177/2325958219867316. 10.1177/2325958219867316 PMC6900587 31389287

[13] Wilson IB, Lee Y, Michaud J, Fowler FJ Jr, Rogers WH. Validation of a new three-item self-report measure for medication adherence. AIDS Behav. 2016;20(11):2700–8. 10.1007/s10461-016-1406-x. 10.1007/s10461-016-1406-x PMC5071118 27098408

[14] Paydary K, Ekhtiari H, Noori M, Rad MV, Hajiabdolbaghi M, SeyedAlinaghi S (2015). Evaluation of the association between Addiction Severity Index and depression with adherence to anti-retroviral therapy among HIV infected patients. Infect Disord Drug Targets.

[15] Olds PK, Kiwanuka JP, Nansera D, Huang Y, Bacchetti P, Jin C (2015). Assessment of HIV antiretroviral therapy adherence by measuring drug concentrations in hair among children in rural Uganda. AIDS Care.

[16] Kelly JD, Hubenthal EA, Lurton G, Empson SF, Barrie MB, Kargbo B (2013). Multiple self-report measures of antiretroviral adherence correlated in Sierra Leone, but did they agree?. Int J STD AIDS.

[17] Buscher A, Hartman C, Kallen MA, Giordano TP (2011). Validity of self-report measures in assessing antiretroviral adherence of newly diagnosed, HAART-naïve. HIV patients HIV Clin Trials.

[18] Haberer JE, Kahane J, Kigozi I, Emenyonu N, Hunt P, Martin J (2010). Real-time adherence monitoring for HIV antiretroviral therapy. AIDS Behav.

[19] Kalichman SC, Amaral CM, Swetzes C, Jones M, Macy R, Kalichman MO (2009). A simple single-item rating scale to measure medication adherence: further evidence for convergent validity. J Int Assoc Physicians AIDS Care (Chic).

[20] Byakika-Tusiime J, Crane J, Oyugi JH, Ragland K, Kawuma A, Musoke P (2009). Longitudinal antiretroviral adherence in HIV+ Ugandan parents and their children initiating HAART in the MTCT-Plus family treatment model: role of depression in declining adherence over time. AIDS Behav.

[21] Muñoz-Moreno JA, Fumaz CR, Ferrer MJ, Tuldrà A, Rovira T, Viladrich C (2007). Assessing self-reported adherence to HIV therapy by questionnaire: the SERAD (Self-Reported Adherence) Study. AIDS Res Hum Retroviruses.

[22] Giordano TP, Guzman D, Clark R, Charlebois ED, Bangsberg DR (2004). Measuring adherence to antiretroviral therapy in a diverse population using a visual analogue scale. HIV Clin Trials.

[23] Walsh JC, Mandalia S, Gazzard BG (2002). Responses to a 1 month self-report on adherence to antiretroviral therapy are consistent with electronic data and virological treatment outcome. AIDS.

[24] Arnsten JH, Demas PA, Farzadegan H, Grant RW, Gourevitch MN, Chang CJ (2001). Antiretroviral therapy adherence and viral suppression in HIV-infected drug users: comparison of self-report and electronic monitoring. Clin Infect Dis.

[25] Wall TL, Sorensen JL, Batki SL, Delucchi KL, London JA, Chesney MA (1995). Adherence to zidovudine (AZT) among HIV-infected methadone patients: A pilot study of supervised therapy and dispensing compared to usual care. Drug Alcohol Depend.

[26] Wu Y, Liu S, Chu L, Zhang Q, Yang J, Qiao S, et al. Hair Zidovudine concentrations predict virologic outcomes among people living with HIV/AIDS in China. Patient Prefer Adherence. 2022;16:1885–96. 10.2147/ppa.S371623. 10.2147/PPA.S371623 PMC9357394 35945983

[27] Ngowi KM, Minja L, Boer IMS, Aarnoutse RE, Masika L, Sprangers MAG (2022). Predicting viral load suppression by self-reported adherence, pharmacy refill counts and real time medication monitoring among people living with HIV in Tanzania. AIDS Res Ther.

[28] Biney IJK, Kyei KA, Ganu VJ, Kenu E, Puplampu P, Manortey S (2021). Antiretroviral therapy adherence and viral suppression among HIV-infected adolescents and young adults at a tertiary hospital in Ghana. Afr J AIDS Res.

[29] Zhang Q, Li X, Qiao S, Shen Z, Zhou Y (2020). Comparing self-reported medication adherence measures with hair antiretroviral concentration among people living with HIV in Guangxi, China. AIDS Res Ther.

[30] Saberi P, Chakravarty D, Ming K, Legnitto D, Gandhi M, Johnson MO, et al. Moving antiretroviral adherence assessments to the modern era: correlations among three novel measures of adherence. AIDS Behav. 2020;24(1):284–90. 10.1007/s10461-019-02744-w. 10.1007/s10461-019-02744-w PMC6996539 31758349

[31] Aduloju OP, Aduloju T, Ade-Ojo IP, Akintayo AA (2020). Medication adherence in hiv-positive pregnant women on antiretroviral therapy attending antenatal clinics in ado metropolis, south-west nigeria: A multicentre study. South African Journal of Obstetrics and Gynaecology.

[32] van Elsland SL, Peters RPH, Grobbelaar N, Ketelo P, Kok MO, Cotton MF, et al. Paediatric ART adherence in South Africa: a comprehensive analysis. AIDS Behav. 2019;23(2):475–88. 10.1007/s10461-018-2235-x. 10.1007/s10461-018-2235-x 30054766

[33] van Elsland SL, Peters RPH, Kok MO, van Toorn R, Springer P, Cotton MF (2018). A treatment-support intervention evaluated in South African paediatric populations with HIV infection or tuberculous meningitis. Trop Med Int Health.

[34] Vale FC, Santa-Helena ET, Santos MA, Carvalho W, Menezes PR, Basso CR (2018). Development and validation of the WebAd-Q Questionnaire to monitor adherence to HIV therapy. Rev Saude Publica.

[35] Sangeda RZ, Mosha F, Aboud S, Kamuhabwa A, Chalamilla G, Vercauteren J (2018). Predictors of non adherence to antiretroviral therapy at an urban HIV care and treatment center in Tanzania. Drug Healthc Patient Saf.

[36] Mudhune V, Gvetadze R, Girde S, Ndivo R, Angira F, Zeh C (2018). Correlation of Adherence by Pill Count, Self-report, MEMS and Plasma Drug Levels to Treatment Response Among Women Receiving ARV Therapy for PMTCT in Kenya. AIDS Behav.

[37] Iwuji C, McGrath N, Calmy A, Dabis F, Pillay D, Newell ML (2018). Universal test and treat is not associated with sub-optimal antiretroviral therapy adherence in rural South Africa: the ANRS 12249 TasP trial. J Int AIDS Soc.

[38] Orrell C, Cohen K, Leisegang R, Bangsberg DR, Wood R, Maartens G (2017). Comparison of six methods to estimate adherence in an ART-naïve cohort in a resource-poor setting: which best predicts virological and resistance outcomes?. AIDS Res Ther.

[39] Mekuria LA, Prins JM, Yalew AW, Sprangers MA, Nieuwkerk PT (2017). Sub-optimal adherence to combination anti-retroviral therapy and its associated factors according to self-report, clinician-recorded and pharmacy-refill assessment methods among HIV-infected adults in Addis Ababa. AIDS Care.

[40] Masa R, Chowa G, Nyirenda V (2017). Barriers and facilitators of antiretroviral therapy adherence in rural Eastern province, Zambia: the role of household economic status. Afr J AIDS Res.

[41] Kioko MT, Pertet AM (2017). Factors contributing to antiretroviral drug adherence among adults living with HIV or AIDS in a Kenyan rural community. Afr J Prim Health Care Fam Med.

[42] Alcaide ML, Ramlagan S, Rodriguez VJ, Cook R, Peltzer K, Weiss SM (2017). Self-report and dry blood spot measurement of antiretroviral medications as markers of adherence in pregnant women in rural South Africa. AIDS Behav.

[43] Rhead R, Masimirembwa C, Cooke G, Takaruza A, Nyamukapa C, Mutsimhi C (2016). Might ART adherence estimates be improved by combining biomarker and self-report data?. PLoS ONE.

[44] Prasitsuebsai W, Kerr SJ, Truong KH, Ananworanich J, Do VC, Nguyen LV (2015). Using lopinavir concentrations in hair samples to assess treatment outcomes on second-line regimens among Asian children. AIDS Res Hum Retroviruses.

[45] Pahari S, Roy S, Mandal A, Kuila S, Panda S (2015). Adherence to anti-retroviral therapy & factors associated with it: A community based cross-sectional study from West Bengal. India Indian J Med Res.

[46] Nsheha AH, Dow DE, Kapanda GE, Hamel BC, Msuya LJ (2014). Adherence to antiretroviral therapy among HIV-infected children receiving care at Kilimanjaro Christian Medical Centre (KCMC), Northern Tanzania: A cross- sectional analytical study. Pan Afr Med J.

[47] Chimhuya S, Nathoo KJ, Rusakaniko S (2013). Non-adherence to highly active antiretroviral therapy in children attending HIV treatment clinic at harare Children's Hospital. Zimbabwe Cent Afr J Med.

[48] Wiens MO, MacLeod S, Musiime V, Ssenyonga M, Kizza R, Bakeera-Kitaka S (2012). Adherence to antiretroviral therapy in HIV-positive adolescents in Uganda assessed by multiple methods: a prospective cohort study. Paediatr Drugs.

[49] Thirumurthy H, Siripong N, Vreeman RC, Pop-Eleches C, Habyarimana JP, Sidle JE (2012). Differences between self-reported and electronically monitored adherence among patients receiving antiretroviral therapy in a resource-limited setting. AIDS.

[50] Nichols SL, Montepiedra G, Farley JJ, Sirois PA, Malee K, Kammerer B (2012). Cognitive, academic, and behavioral correlates of medication adherence in children and adolescents with perinatally acquired HIV infection. J Dev Behav Pediatr.

[51] Musiime V, Kayiwa J, Kiconco M, Tamale W, Alima H, Mugerwa H (2012). Response to antiretroviral therapy of HIV type 1-infected children in urban and rural settings of Uganda. AIDS Res Hum Retroviruses.

[52] Gutierrez EB, Sartori AM, Schmidt AL, Piloto BM, França BB, de Oliveira AS (2012). Measuring adherence to antiretroviral treatment: the role of pharmacy records of drug withdrawals. AIDS Behav.

[53] da Costa TM, Barbosa BJ, Gomes e Costa DA, Sigulem D, de Fátima Marin H, Filho AC, et al. Results of a randomized controlled trial to assess the effects of a mobile SMS-based intervention on treatment adherence in HIV/AIDS-infected Brazilian women and impressions and satisfaction with respect to incoming messages. Int J Med Inform. 2012;81(4):257–69; 10.1016/j.ijmedinf.2011.10.002. 10.1016/j.ijmedinf.2011.10.002 PMC3766367 22296762

[54] Senkomago V, Guwatudde D, Breda M, Khoshnood K (2011). Barriers to antiretroviral adherence in HIV-positive patients receiving free medication in Kayunga. Uganda AIDS Care.

[55] Rocha GM, Machado CJ, Acurcio Fde A, Guimarães MD (2011). Monitoring adherence to antiretroviral treatment in Brazil: an urgent challenge. Cad Saude Publica.

[56] Ndubuka NO, Ehlers VJ (2011). Adult patients' adherence to anti-retroviral treatment: a survey correlating pharmacy refill records and pill counts with immunological and virological indices. Int J Nurs Stud.

[57] Kunutsor S, Evans M, Thoulass J, Walley J, Katabira E, Newell JN (2010). Ascertaining baseline levels of antiretroviral therapy adherence in Uganda: a multimethod approach. J Acquir Immune Defic Syndr.

[58] Skrajner MJ, Camp CJ, Haberman JL, Heckman TG, Kochman A, Frentiu C (2009). Use of Videophone Technology to Address Medication Adherence Issues in Persons with HIV. HIV AIDS (Auckl).

[59] Rougemont M, Stoll BE, Elia N, Ngang P (2009). Antiretroviral treatment adherence and its determinants in Sub-Saharan Africa: a prospective study at Yaounde Central Hospital. Cameroon AIDS Res Ther.

[60] Minzi OM, Naazneen AS (2008). Validation of self-report and hospital pill count using unannounced home pill count as methods for determination of adherence to antiretroviral therapy. Tanzan J Health Res.

[61] Lu M, Safren SA, Skolnik PR, Rogers WH, Coady W, Hardy H (2008). Optimal recall period and response task for self-reported HIV medication adherence. AIDS Behav.

[62] Kerr T, Hogg RS, Yip B, Tyndall MW, Montaner J, Wood E (2008). Validity of self-reported adherence among injection drug users. J Int Assoc Physicians AIDS Care (Chic).

[63] Vriesendorp R, Cohen A, Kristanto P, Vrijens B, Rakesh P, Anand B (2007). Adherence to HAART therapy measured by electronic monitoring in newly diagnosed HIV patients in Botswana. Eur J Clin Pharmacol.

[64] Vaz MJ, Barros SM, Palacios R, Senise JF, Lunardi L, Amed AM (2007). HIV-infected pregnant women have greater adherence with antiretroviral drugs than non-pregnant women. Int J STD AIDS.

[65] Plipat N, Kottapat U, Komoltri C, Voradilokkul J, Anansakunwatt W, Chearskul P (2007). Evaluation of a practical method to assess antiretroviral adherence in HIV-infected Thai children. Southeast Asian J Trop Med Public Health.

[66] Bell DJ, Kapitao Y, Sikwese R, van Oosterhout JJ, Lalloo DG (2007). Adherence to antiretroviral therapy in patients receiving free treatment from a government hospital in Blantyre. Malawi J Acquir Immune Defic Syndr.

[67] Llabre MM, Weaver KE, Durán RE, Antoni MH, McPherson-Baker S, Schneiderman N (2006). A measurement model of medication adherence to highly active antiretroviral therapy and its relation to viral load in HIV-positive adults. AIDS Patient Care STDS.

[68] Holzemer WL, Bakken S, Portillo CJ, Grimes R, Welch J, Wantland D (2006). Testing a nurse-tailored HIV medication adherence intervention. Nurs Res.

[69] Wohl DA, Stephenson BL, Golin CE, Kiziah CN, Rosen D, Ngo B (2003). Adherence to directly observed antiretroviral therapy among human immunodeficiency virus-infected prison inmates. Clin Infect Dis.

[70] Murphy DA, Greenwell L, Hoffman D (2002). Factors associated with antiretroviral adherence among HIV-infected women with children. Women Health.

[71] Frick PA, Gal P, Lane TW, Sewell PC (1998). Antiretroviral medication compliance in patients with AIDS. AIDS Patient Care STDS.

[72] El Alili M, Vrijens B, Demonceau J, Evers SM, Hiligsmann M (2016). A scoping review of studies comparing the medication event monitoring system (MEMS) with alternative methods for measuring medication adherence. Br J Clin Pharmacol.

[73] Rivers PH, Ardagh-Walter N, Wright EC (1998). Measurement of anticonvulsant adherence behaviour in the community using a Medication Events Monitoring System (MEMS). Health Care Anal.

[74] Stirratt MJ, Dunbar-Jacob J, Crane HM, Simoni JM, Czajkowski S, Hilliard ME (2015). Self-report measures of medication adherence behavior: recommendations on optimal use. Transl Behav Med.

[75] Kabore L, Muntner P, Chamot E, Zinski A, Burkholder G, Mugavero MJ (2015). Self-report measures in the assessment of antiretroviral medication adherence: comparison with medication possession ratio and HIV viral load. J Int Assoc Provid AIDS Care.

[76] UNAIDS. Documents. (2024). Accessed 27 Feb 2024.

[77] Williams AB, Amico KR, Bova C, Womack JA (2013). A proposal for quality standards for measuring medication adherence in research. AIDS Behav.

[78] Wagner G, Miller LG (2004). Is the influence of social desirability on patients' self-reported adherence overrated?. J Acquir Immune Defic Syndr.

[79] Smith R, Villanueva G, Probyn K, Sguassero Y, Ford N, Orrell C, et al. Accuracy of measures for antiretroviral adherence in people living with HIV. Cochrane Database Syst Rev. 2022;7(7):Cd013080; 10.1002/14651858.CD013080.pub2. 10.1002/14651858.CD013080.pub2 PMC9309033 35871531

[80] Che Pa MF, Makmor-Bakry M, Islahudin F. Digital health in enhancing antiretroviral therapy adherence: a systematic review and meta-analysis. AIDS Patient Care STDS. 2023;37(11):507–16. 10.1089/apc.2023.0170. 10.1089/apc.2023.0170 37956244

