# Supplementary material for: Discrepancies between self-reported medication in adherence and indirect measurement adherence among patients undergoing antiretroviral therapy: a systematic review
Source: Infect Dis Poverty. 2024 Jul 5;13:51. doi: 10.1186/s40249-024-01221-4 (PMC11225374; doi:10.1186/s40249-024-01221-4)
Supplement: Supplementary file 1 — Supplementary Material 1. [file 40249_2024_1221_MOESM1_ESM.docx]

****Pubmed N=318****

|  | Search Terms |
| --- | --- |
| #1 | **((((((self report[MeSH Terms]) OR (self report*[Title/Abstract])) OR (patient report*[Title/Abstract])) OR (questionaire*[Title/Abstract])) OR (scale*[Title/Abstract])) OR (visual analog scale[MeSH Terms]))** |
| **#2** | **((((medication adherence[MeSH Terms]) OR (patient compliance[MeSH Terms])) OR (adherence*[Title/Abstract])) OR (compliance*[Title/Abstract]) ) OR (treatment completion[Title/Abstract])** |
| **#3** | **((((((((((MEMS[Title/Abstract]) OR (****pill count*[Title/Abstract])) OR (pharmacy refill[Title/Abstract])) OR (dried blood[Title/Abstract])) OR (medical event monitoring system[Title/Abstract]) ) OR (urine[Title/Abstract])) OR (Directly Observed Therapy[MeSH Terms]) ) OR (medical records[MeSH Terms])) OR (electronic medication*[Title/Abstract])) OR (hair[Title/Abstract])) OR (bio*[Title/Abstract])** |
| **#4** | **(((antiretroviral therapy, highly active[MeSH Terms]) OR (HIV[MeSH Terms])) OR (acquired immune deficiency syndrome[MeSH Terms])) OR ("antiretroviral"[Title/Abstract])** |
| #5 | #1 AND #2 AND #3 AND #4=318 |

**Embase N=269**

|  | Search Terms |
| --- | --- |
| #1 | 'self report':ti,ab,kw OR 'patient report':ti,ab,kw OR 'self reported':ti,ab,kw OR 'patient reported':ti,ab,kw OR questionaire:ti,ab,kw OR scale:ti,ab,kw |
| #2 | adherence:ti,ab,kw OR compliance:ti,ab,kw OR completion:ti,ab,kw OR nonadherence:ti,ab,kw |
| #3 | mems:ti,ab,kw OR 'medical event mornitoring system':ti,ab,kw OR 'medical record':ti,ab,kw OR 'pill count':ti,ab,kw OR 'dried blood':ti,ab,kw OR 'pharmacy refill':ti,ab,kw OR 'electronic medication':ti,ab,kw OR urine:ti,ab,kw OR 'directly observed':ti,ab,kw OR biomaker:ti,ab,kw OR hair:ti,ab,kw |
| #4 | 'antiretroviral therapy':ti,ab,kw |
| #5 | #1 AND #2 AND #3 AND #4 |
| #6 | 'antiretroviral therapy':ti,ab,kw OR 'human immunodeficiency virus':ti,ab,kw OR 'acquired immune deficiency syndrome':ti,ab,kw |
| #7 | #1 AND #2 AND #3 AND #6 |

**Cochrane N=21**

Reviews matching adherence in Title Abstract Keyword AND antiretroviral in Title Abstract Keyword - (Word variations have been searched)
