# Supplementary material for: Discrepancies between self-reported medication in adherence and indirect measurement adherence among patients undergoing antiretroviral therapy: a systematic review
Source: Infect Dis Poverty. 2024 Jul 5;13:51. doi: 10.1186/s40249-024-01221-4 (PMC11225374; doi:10.1186/s40249-024-01221-4)
Supplement: Supplementary file 2 — Supplementary Material 2. [file 40249_2024_1221_MOESM2_ESM.docx]

| Study | Q1 | Q2 | Q3 | Q4 | Q5 | Q6 | Q7 | Q8 | Q9 | Q10 | Q11 | 总分 | 评级 |
| --- | --- | --- | --- | --- | --- | --- | --- | --- | --- | --- | --- | --- | --- |
| Wu2022[1] | 1 | 1 | 1 | 1 | 1 | 1 | 1 | 1 | 0 | 1 | 1 | 10 | High |
| Ngowi2022[2] | 1 | 1 | 1 | 1 | 1 | 1 | 1 | 0 | 1 | 1 | 1 | 10 | High |
| Kizindo2022[3] | 1 | 0 | 1 | 1 | 1 | 1 | 0 | 0 | 1 | 1 | 1 | 8 | High |
| Abiodun2021[4] | 1 | 1 | 1 | 0 | 1 | 1 | 1 | 1 | 1 | 1 | 1 | 10 | High |
| Biney2021[5] | 1 | 1 | 1 | 1 | 1 | 1 | 1 | 0 | 1 | 1 | 1 | 10 | High |
| Aduloju2020[6] | 1 | 1 | 1 | 1 | 1 | 1 | 1 | 0 | 0 | 1 | 1 | 9 | High |
| Allam2020[7] | 1 | 1 | 1 | 1 | 1 | 0 | 1 | 1 | 1 | 1 | / | 9 | High |
| Peng2020[8] | 1 | 1 | 1 | 1 | 1 | 1 | 1 | 1 | 0 | 1 | 1 | 10 | High |
| Saberi2020[9] | 1 | 1 | 1 | 1 | 1 | 1 | 1 | 1 | 1 | 1 | 1 | 11 | High |
| Zhang2020[10] | 1 | 1 | 1 | 1 | 1 | 1 | 1 | 1 | 1 | 1 | 1 | 11 | High |
| Gaifer2019[11] | 1 | 1 | 1 | 1 | 1 | 1 | 1 | 0 | 1 | 1 | 1 | 10 | High |
| Van2019[12] | 1 | 1 | 1 | 1 | 1 | 1 | 1 | 1 | 1 | 1 | / | 10 | High |
| Lwuji2018[13] | 1 | 1 | 1 | 1 | 1 | 1 | 1 | 1 | 1 | 1 | 1 | 11 | High |
| Mudhune2018[14] | 1 | 1 | 1 | 1 | 1 | 1 | 1 | 1 | 1 | 1 | 1 | 11 | High |
| Sangeda2018[15] | 1 | 1 | 1 | 1 | 1 | 1 | 1 | 0 | 1 | 1 | 1 | 10 | High |
| vale2018[16] | 1 | 1 | 1 | 1 | 1 | 1 | 1 | 1 | 1 | 1 | 1 | 11 | High |
| Van elsland2018[17] | 1 | 1 | 1 | 1 | 1 | 1 | 1 | 1 | 1 | 1 | 1 | 11 | High |
| Alcaide2017[18] | 1 | 1 | 1 | 1 | 1 | 1 | 0 | 0 | 1 | 1 | 1 | 9 | High |
| Kioko2017[19] | 1 | 1 | 1 | 1 | 1 | 1 | 0 | 0 | 1 | 1 | / | 8 | High |
| Masa2017[20] | 1 | 0 | 1 | 1 | 1 | 0 | 1 | 0 | 1 | 1 | 1 | 8 | High |
| Mekuria2017[21] | 1 | 1 | 1 | 1 | 1 | 1 | 1 | 0 | 1 | 1 | 1 | 10 | High |
| Orrell2017[22] | 1 | 1 | 1 | 1 | 1 | 1 | 1 | 0 | 1 | 1 | 1 | 10 | High |
| Wilson2016[23] | 1 | 1 | 1 | 1 | 1 | 1 | 0 | 1 | 1 | 1 | 1 | 10 | High |
| Rhead2016[24] | 1 | 1 | 1 | 1 | 1 | 1 | 0 | 0 | 0 | 1 | 1 | 8 | High |
| Olds2015[25] | 1 | 1 | 1 | 1 | 1 | 1 | 1 | 0 | 1 | 1 | 1 | 10 | High |
| Pahari2015[26] | 1 | 1 | 1 | 1 | 1 | 0 | 0 | 0 | 1 | 1 | 1 | 8 | High |
| Paydary2015[27] | 1 | 1 | 1 | 1 | 1 | 1 | 1 | 1 | 1 | 1 | 1 | 11 | High |
| Prasitsuebsai2015[28] | 1 | 1 | 1 | 0 | 1 | 1 | 0 | 1 | 1 | 1 | 1 | 9 | High |
| Nsheha2014[29] | 1 | 1 | 1 | 1 | 1 | 0 | 1 | 1 | 0 | 1 | / | 8 | High |
| Kelly2013[30] | 1 | 1 | 1 | 1 | 1 | 1 | 1 | 0 | 1 | 1 | 1 | 10 | High |
| Chimhuya2013[31] | 1 | 1 | 1 | 1 | 1 | 0 | 1 | 0 | 1 | 1 | / | 8 | High |
| Da costa2012[32] | 1 | 1 | 1 | 1 | 1 | 1 | 1 | 1 | 1 | 1 | 1 | 11 | High |
| Gutierrez2012[33] | 1 | 1 | 1 | 1 | 1 | 0 | 1 | 0 | 1 | 1 | / | 8 | High |
| Muslime2012[34] | 1 | 1 | 1 | 1 | 1 | 0 | 0 | 1 | 1 | 1 | 1 | 9 | High |
| Nichols2012[35] | 1 | 1 | 1 | 1 | 1 | 1 | 1 | 1 | 1 | 1 | 1 | 11 | High |
| Thirumurthy2012[36] | 1 | 1 | 1 | 1 | 1 | 1 | 1 | 0 | 1 | 1 | 1 | 10 | High |
| wiens2012[37] | 1 | 1 | 1 | 1 | 1 | 1 | 1 | 1 | 1 | 1 | 1 | 11 | High |
| Ndubuka2011[38] | 1 | 1 | 1 | 1 | 1 | 1 | 1 | 0 | 1 | 0 | / | 8 | High |
| Rocha2011[39] | 1 | 1 | 1 | 1 | 1 | 1 | 1 | 0 | 1 | 1 | 1 | 10 | High |
| Senkomaga2011[40] | 1 | 1 | 1 | 1 | 1 | 1 | 1 | 0 | 1 | 1 | 1 | 10 | High |
| Kunutsor2010[41] | 1 | 1 | 1 | 0 | 1 | 1 | 1 | 0 | 1 | 1 | 1 | 9 | High |
| Haberer2010[42] | 1 | 1 | 1 | 1 | 1 | 1 | 1 | 0 | 1 | 1 | 1 | 10 | High |
| Buscher2010[43] | 1 | 1 | 1 | 1 | 1 | 1 | 0 | 0 | 1 | 1 | 1 | 9 | High |
| Byakika2009[44] | 1 | 1 | 1 | 1 | 1 | 1 | 0 | 1 | 0 | 1 | 0 | 8 | High |
| Kalichman2009[45] | 1 | 1 | 1 | 1 | 1 | 1 | 0 | 0 | 0 | 1 | 1 | 8 | High |
| Rougemont2009[46] | 1 | 1 | 1 | 1 | 1 | 1 | 1 | 0 | 1 | 1 | 1 | 10 | High |
| Skrajner2009[47] | 1 | 1 | 1 | 0 | 1 | 1 | 1 | 1 | 1 | 1 | 1 | 10 | High |
| Kerr2008[48] | 1 | 0 | 1 | 1 | 1 | 1 | 0 | 1 | 0 | 1 | 1 | 8 | High |
| Lu2008[49] | 1 | 0 | 1 | 1 | 1 | 1 | 0 | 1 | 0 | 1 | 1 | 8 | High |
| Minzi2008[50] | 1 | 1 | 0 | 1 | 1 | 1 | 0 | 1 | 0 | 1 | 1 | 8 | High |
| Nabukeera-Barungi2007[51] | 1 | 1 | 1 | 1 | 1 | 1 | 0 | 1 | 0 | 1 | 1 | 9 | High |
| Bell2007[52] | 1 | 1 | 1 | 1 | 1 | 1 | 0 | 0 | 0 | 1 | 1 | 8 | High |
| Muñoz-Moreno2007[53] | 1 | 0 | 1 | 0 | 1 | 1 | 0 | 1 | 1 | 1 | 1 | 8 | High |
| Vriesendorp2007[54] | 1 | 0 | 1 | 1 | 1 | 1 | 1 | 0 | 1 | 1 | 1 | 9 | High |
| Plipat2007[55] | 1 | 1 | 1 | 1 | 1 | 1 | 1 | 0 | 1 | 1 | 1 | 10 | High |
| Vaz2007[56] | 1 | 1 | 1 | 1 | 1 | 1 | 1 | 1 | 1 | 1 | 1 | 11 | High |
| Holzemer2006[57] | 1 | 1 | 0 | 1 | 1 | 1 | 0 | 1 | 0 | 1 | 1 | 8 | High |
| Llabre2006[58] | 1 | 1 | 1 | 1 | 1 | 1 | 1 | 1 | 0 | 0 | / | 8 | High |
| Giordano2004[59] | 1 | 1 | 1 | 1 | 1 | 1 | 0 | 0 | 1 | 1 | 1 | 9 | High |
| Wohl2003[60] | 1 | 1 | 1 | 1 | 1 | 1 | 1 | 0 | 1 | 1 | 1 | 10 | High |
| Walsh2002[61] | 1 | 1 | 1 | 1 | 1 | 1 | 1 | 0 | 1 | 1 | 1 | 10 | High |
| Murphy2002[62] | 1 | 1 | 1 | 1 | 1 | 1 | 0 | 0 | 1 | 1 | 1 | 9 | High |
| Arnsten2001[63] | 1 | 1 | 1 | 1 | 1 | 1 | 1 | 0 | 1 | 1 | 1 | 10 | High |
| Frick1998[64] | 1 | 1 | 1 | 1 | 1 | 1 | 1 | 0 | 1 | 1 | / | 9 | High |
| Wall1995[65] | 1 | 1 | 1 | 1 | 1 | 1 | 0 | 0 | 1 | 1 | 0 | 8 | High |

Note:

Q 1: Define the source of information (Survey, record review)

Q 2: List inclusion and exclusion criteria for subjects or refer to previous publications

Q 3: Indicate time period used for identifying subjects

Q 4: Indicate whether subjects were consecutive if not population based. Whether subjects are representative of the average in the community?

Q 5: Indicate if evaluators of subjective components of study were masked to other aspects of the status of the participants. Are the evaluators professional (trained /calibrated)?

Q 6: Describe any assessments undertaken for quality assurance purposes (e.g., test/retest of primary outcome measurements)

Q 7: If any, explain any subject exclusions from analysis

Q 8: Describe how confounding was assessed and/or controlled.

Q 9: If possible, explain how lost data is handled in the analysis

Q 10: Summarize patient response rates and completeness of data collection

Q 11: Identify the percentage of expected incomplete patient data or follow-up results

Criteria: 1=clearly mentioned; 0=not done or not mentioned or unclear information; /=not applicable. Studies with scores≥8 were considered with high quality.

1. Wu Y, Liu S, Chu L, Zhang Q, Yang J, Qiao S, et al. Hair Zidovudine Concentrations Predict Virologic Outcomes Among People Living with HIV/AIDS in China. Patient Prefer Adherence. 2022;16:1885-96; doi: 10.2147/ppa.S371623.

2. Ngowi KM, Minja L, Boer IMS, Aarnoutse RE, Masika L, Sprangers MAG, et al. Predicting viral load suppression by self-reported adherence, pharmacy refill counts and real time medication monitoring among people living with HIV in Tanzania. AIDS Res Ther. 2022;19(1):51; doi: 10.1186/s12981-022-00475-y.

3. Kizindo J, Marealle AI, Mutagonda R, Mlyuka HJ, Mikomangwa WP, Kilonzi M, et al. Adherence to Antiretroviral Therapy Among HIV-Infected Clients Attending Opioid Treatment Program Clinics in Dar es Salaam, Tanzania. Cureus. 2022;14(5):e25522; doi: 10.7759/cureus.25522.

4. Abiodun O, Ladi-Akinyemi B, Olu-Abiodun O, Sotunsa J, Bamidele F, Adepoju A, et al. A Single-Blind, Parallel Design RCT to Assess the Effectiveness of SMS Reminders in Improving ART Adherence Among Adolescents Living with HIV (STARTA Trial). J Adolesc Health. 2021;68(4):728-36; doi: 10.1016/j.jadohealth.2020.11.016.

5. Biney IJK, Kyei KA, Ganu VJ, Kenu E, Puplampu P, Manortey S, et al. Antiretroviral therapy adherence and viral suppression among HIV-infected adolescents and young adults at a tertiary hospital in Ghana. Afr J AIDS Res. 2021;20(4):270-6; doi: 10.2989/16085906.2021.1998783.

6. Aduloju OP, Aduloju T, Ade-Ojo IP, Akintayo AA. Medication adherence in hiv-positive pregnant women on antiretroviral therapy attending antenatal clinics in ado metropolis, south-west nigeria: A multicentre study. South African Journal of Obstetrics and Gynaecology. 2020;26(2):1-6; doi: <https://dx.doi.org/10.7196/sajog.1611>.

7. Allam RR, Takamiya M, Pant R, Gandham S, Yeldandi VV, Thomas J, et al. Factors associated with non-adherence to antiretroviral therapy among female sex workers living with HIV in Hyderabad, India. Int J STD AIDS. 2020;31(8):735-46; doi: 10.1177/0956462420920145.

8. Pang Y, Molton JS, Ooi WT, Paton NI, He HG. Preliminary Effects of a Mobile Interactive Supervised Therapy Intervention on People Living With HIV: Pilot Randomized Controlled Trial. JMIR Mhealth Uhealth. 2020;8(3):e15702; doi: 10.2196/15702.

9. Saberi P, Chakravarty D, Ming K, Legnitto D, Gandhi M, Johnson MO, et al. Moving Antiretroviral Adherence Assessments to the Modern Era: Correlations Among Three Novel Measures of Adherence. AIDS Behav. 2020;24(1):284-90; doi: 10.1007/s10461-019-02744-w.

10. Zhang Q, Li X, Qiao S, Shen Z, Zhou Y. Comparing self-reported medication adherence measures with hair antiretroviral concentration among people living with HIV in Guangxi, China. AIDS Res Ther. 2020;17(1):8; doi: 10.1186/s12981-020-00265-4.

11. Gaifer Z, Boulassel MR. Comparative Analysis of two Methods of Measuring Antiretroviral Therapy Adherence in HIV-Infected Omani Patients. J Int Assoc Provid AIDS Care. 2019;18:2325958219867316; doi: 10.1177/2325958219867316.

12. van Elsland SL, Peters RPH, Grobbelaar N, Ketelo P, Kok MO, Cotton MF, et al. Paediatric ART Adherence in South Africa: A Comprehensive Analysis. AIDS Behav. 2019;23(2):475-88; doi: 10.1007/s10461-018-2235-x.

13. Iwuji C, McGrath N, Calmy A, Dabis F, Pillay D, Newell ML, et al. Universal test and treat is not associated with sub-optimal antiretroviral therapy adherence in rural South Africa: the ANRS 12249 TasP trial. J Int AIDS Soc. 2018;21(6):e25112; doi: 10.1002/jia2.25112.

14. Mudhune V, Gvetadze R, Girde S, Ndivo R, Angira F, Zeh C, et al. Correlation of Adherence by Pill Count, Self-report, MEMS and Plasma Drug Levels to Treatment Response Among Women Receiving ARV Therapy for PMTCT in Kenya. AIDS Behav. 2018;22(3):918-28; doi: 10.1007/s10461-017-1724-7.

15. Sangeda RZ, Mosha F, Aboud S, Kamuhabwa A, Chalamilla G, Vercauteren J, et al. Predictors of non adherence to antiretroviral therapy at an urban HIV care and treatment center in Tanzania. Drug Healthc Patient Saf. 2018;10:79-88; doi: 10.2147/dhps.S143178.

16. Vale FC, Santa-Helena ET, Santos MA, Carvalho W, Menezes PR, Basso CR, et al. Development and validation of the WebAd-Q Questionnaire to monitor adherence to HIV therapy. Rev Saude Publica. 2018;52:62; doi: 10.11606/s1518-8787.2018052000337.

17. van Elsland SL, Peters RPH, Kok MO, van Toorn R, Springer P, Cotton MF, et al. A treatment-support intervention evaluated in South African paediatric populations with HIV infection or tuberculous meningitis. Trop Med Int Health. 2018;23(10):1129-40; doi: 10.1111/tmi.13134.

18. Alcaide ML, Ramlagan S, Rodriguez VJ, Cook R, Peltzer K, Weiss SM, et al. Self-Report and Dry Blood Spot Measurement of Antiretroviral Medications as Markers of Adherence in Pregnant Women in Rural South Africa. AIDS Behav. 2017;21(7):2135-40; doi: 10.1007/s10461-017-1760-3.

19. Kioko MT, Pertet AM. Factors contributing to antiretroviral drug adherence among adults living with HIV or AIDS in a Kenyan rural community. Afr J Prim Health Care Fam Med. 2017;9(1):e1-e7; doi: 10.4102/phcfm.v9i1.1343.

20. Masa R, Chowa G, Nyirenda V. Barriers and facilitators of antiretroviral therapy adherence in rural Eastern province, Zambia: the role of household economic status. Afr J AIDS Res. 2017;16(2):91-9; doi: 10.2989/16085906.2017.1308386.

21. Mekuria LA, Prins JM, Yalew AW, Sprangers MA, Nieuwkerk PT. Sub-optimal adherence to combination anti-retroviral therapy and its associated factors according to self-report, clinician-recorded and pharmacy-refill assessment methods among HIV-infected adults in Addis Ababa. AIDS Care. 2017;29(4):428-35; doi: 10.1080/09540121.2016.1234681.

22. Orrell C, Cohen K, Leisegang R, Bangsberg DR, Wood R, Maartens G. Comparison of six methods to estimate adherence in an ART-naïve cohort in a resource-poor setting: which best predicts virological and resistance outcomes? AIDS Res Ther. 2017;14(1):20; doi: 10.1186/s12981-017-0138-y.

23. Wilson IB, Lee Y, Michaud J, Fowler FJ, Jr., Rogers WH. Validation of a New Three-Item Self-Report Measure for Medication Adherence. AIDS Behav. 2016;20(11):2700-8; doi: 10.1007/s10461-016-1406-x.

24. Rhead R, Masimirembwa C, Cooke G, Takaruza A, Nyamukapa C, Mutsimhi C, et al. Might ART Adherence Estimates Be Improved by Combining Biomarker and Self-Report Data? PLoS One. 2016;11(12):e0167852; doi: 10.1371/journal.pone.0167852.

25. Olds PK, Kiwanuka JP, Nansera D, Huang Y, Bacchetti P, Jin C, et al. Assessment of HIV antiretroviral therapy adherence by measuring drug concentrations in hair among children in rural Uganda. AIDS Care. 2015;27(3):327-32; doi: 10.1080/09540121.2014.983452.

26. Pahari S, Roy S, Mandal A, Kuila S, Panda S. Adherence to anti-retroviral therapy & factors associated with it: A community based cross-sectional study from West Bengal, India. Indian J Med Res. 2015;142(3):301-10; doi: 10.4103/0971-5916.166595.

27. Paydary K, Ekhtiari H, Noori M, Rad MV, Hajiabdolbaghi M, SeyedAlinaghi S. Evaluation of the association between Addiction Severity Index and depression with adherence to anti-retroviral therapy among HIV infected patients. Infect Disord Drug Targets. 2015;15(3):177-83; doi: 10.2174/1871526515666150928115103.

28. Prasitsuebsai W, Kerr SJ, Truong KH, Ananworanich J, Do VC, Nguyen LV, et al. Using Lopinavir Concentrations in Hair Samples to Assess Treatment Outcomes on Second-Line Regimens Among Asian Children. AIDS Res Hum Retroviruses. 2015;31(10):1009-14; doi: 10.1089/aid.2015.0111.

29. Nsheha AH, Dow DE, Kapanda GE, Hamel BC, Msuya LJ. Adherence to antiretroviral therapy among HIV-infected children receiving care at Kilimanjaro Christian Medical Centre (KCMC), Northern Tanzania: A cross- sectional analytical study. Pan Afr Med J. 2014;17:238; doi: 10.11604/pamj.2014.17.238.2280.

30. Kelly JD, Hubenthal EA, Lurton G, Empson SF, Barrie MB, Kargbo B, et al. Multiple self-report measures of antiretroviral adherence correlated in Sierra Leone, but did they agree? Int J STD AIDS. 2013;24(12):931-7; doi: 10.1177/0956462413487327.

31. Chimhuya S, Nathoo KJ, Rusakaniko S. Non-adherence to highly active antiretroviral therapy in children attending HIV treatment clinic at harare Children's Hospital, Zimbabwe. Cent Afr J Med. 2013;59(9-12):63-70.

32. da Costa TM, Barbosa BJ, Gomes e Costa DA, Sigulem D, de Fátima Marin H, Filho AC, et al. Results of a randomized controlled trial to assess the effects of a mobile SMS-based intervention on treatment adherence in HIV/AIDS-infected Brazilian women and impressions and satisfaction with respect to incoming messages. Int J Med Inform. 2012;81(4):257-69; doi: 10.1016/j.ijmedinf.2011.10.002.

33. Gutierrez EB, Sartori AM, Schmidt AL, Piloto BM, França BB, de Oliveira AS, et al. Measuring adherence to antiretroviral treatment: the role of pharmacy records of drug withdrawals. AIDS Behav. 2012;16(6):1482-90; doi: 10.1007/s10461-012-0168-3.

34. Musiime V, Kayiwa J, Kiconco M, Tamale W, Alima H, Mugerwa H, et al. Response to antiretroviral therapy of HIV type 1-infected children in urban and rural settings of Uganda. AIDS Res Hum Retroviruses. 2012;28(12):1647-57; doi: 10.1089/aid.2011.0313.

35. Nichols SL, Montepiedra G, Farley JJ, Sirois PA, Malee K, Kammerer B, et al. Cognitive, academic, and behavioral correlates of medication adherence in children and adolescents with perinatally acquired HIV infection. J Dev Behav Pediatr. 2012;33(4):298-308; doi: 10.1097/DBP.0b013e31824bef47.

36. Thirumurthy H, Siripong N, Vreeman RC, Pop-Eleches C, Habyarimana JP, Sidle JE, et al. Differences between self-reported and electronically monitored adherence among patients receiving antiretroviral therapy in a resource-limited setting. Aids. 2012;26(18):2399-403; doi: 10.1097/QAD.0b013e328359aa68.

37. Wiens MO, MacLeod S, Musiime V, Ssenyonga M, Kizza R, Bakeera-Kitaka S, et al. Adherence to antiretroviral therapy in HIV-positive adolescents in Uganda assessed by multiple methods: a prospective cohort study. Paediatr Drugs. 2012;14(5):331-5; doi: 10.2165/11599280-000000000-00000.

38. Ndubuka NO, Ehlers VJ. Adult patients' adherence to anti-retroviral treatment: a survey correlating pharmacy refill records and pill counts with immunological and virological indices. Int J Nurs Stud. 2011;48(11):1323-9; doi: 10.1016/j.ijnurstu.2011.04.006.

39. Rocha GM, Machado CJ, Acurcio Fde A, Guimarães MD. Monitoring adherence to antiretroviral treatment in Brazil: an urgent challenge. Cad Saude Publica. 2011;27 Suppl 1:S67-78; doi: 10.1590/s0102-311x2011001300008.

40. Senkomago V, Guwatudde D, Breda M, Khoshnood K. Barriers to antiretroviral adherence in HIV-positive patients receiving free medication in Kayunga, Uganda. AIDS Care. 2011;23(10):1246-53; doi: 10.1080/09540121.2011.564112.

41. Kunutsor S, Evans M, Thoulass J, Walley J, Katabira E, Newell JN, et al. Ascertaining baseline levels of antiretroviral therapy adherence in Uganda: a multimethod approach. J Acquir Immune Defic Syndr. 2010;55(2):221-4; doi: 10.1097/QAI.0b013e3181e255ec.

42. Haberer JE, Kahane J, Kigozi I, Emenyonu N, Hunt P, Martin J, et al. Real-time adherence monitoring for HIV antiretroviral therapy. AIDS Behav. 2010;14(6):1340-6; doi: 10.1007/s10461-010-9799-4.

43. Buscher A, Hartman C, Kallen MA, Giordano TP. Validity of self-report measures in assessing antiretroviral adherence of newly diagnosed, HAART-naïve, HIV patients. HIV Clin Trials. 2011;12(5):244-54; doi: 10.1310/hct1205-244.

44. Byakika-Tusiime J, Crane J, Oyugi JH, Ragland K, Kawuma A, Musoke P, et al. Longitudinal antiretroviral adherence in HIV+ Ugandan parents and their children initiating HAART in the MTCT-Plus family treatment model: role of depression in declining adherence over time. AIDS Behav. 2009;13 Suppl 1:82-91; doi: 10.1007/s10461-009-9546-x.

45. Kalichman SC, Amaral CM, Swetzes C, Jones M, Macy R, Kalichman MO, et al. A simple single-item rating scale to measure medication adherence: further evidence for convergent validity. J Int Assoc Physicians AIDS Care (Chic). 2009;8(6):367-74; doi: 10.1177/1545109709352884.

46. Rougemont M, Stoll BE, Elia N, Ngang P. Antiretroviral treatment adherence and its determinants in Sub-Saharan Africa: a prospective study at Yaounde Central Hospital, Cameroon. AIDS Res Ther. 2009;6:21; doi: 10.1186/1742-6405-6-21.

47. Skrajner MJ, Camp CJ, Haberman JL, Heckman TG, Kochman A, Frentiu C. Use of Videophone Technology to Address Medication Adherence Issues in Persons with HIV. HIV AIDS (Auckl). 2009;1:23-30; doi: 10.2147/hiv.S6325.

48. Kerr T, Hogg RS, Yip B, Tyndall MW, Montaner J, Wood E. Validity of self-reported adherence among injection drug users. J Int Assoc Physicians AIDS Care (Chic). 2008;7(4):157-9; doi: 10.1177/1545109708320686.

49. Lu M, Safren SA, Skolnik PR, Rogers WH, Coady W, Hardy H, et al. Optimal recall period and response task for self-reported HIV medication adherence. AIDS Behav. 2008;12(1):86-94; doi: 10.1007/s10461-007-9261-4.

50. Minzi OM, Naazneen AS. Validation of self-report and hospital pill count using unannounced home pill count as methods for determination of adherence to antiretroviral therapy. Tanzan J Health Res. 2008;10(2):84-8; doi: 10.4314/thrb.v10i2.14350.

51. Nabukeera-Barungi N, Kalyesubula I, Kekitiinwa A, Byakika-Tusiime J, Musoke P. Adherence to antiretroviral therapy in children attending Mulago Hospital, Kampala. Ann Trop Paediatr. 2007;27(2):123-31; doi: 10.1179/146532807x192499.

52. Bell DJ, Kapitao Y, Sikwese R, van Oosterhout JJ, Lalloo DG. Adherence to antiretroviral therapy in patients receiving free treatment from a government hospital in Blantyre, Malawi. J Acquir Immune Defic Syndr. 2007;45(5):560-3; doi: 10.1097/QAI.0b013e3180decadb.

53. Muñoz-Moreno JA, Fumaz CR, Ferrer MJ, Tuldrà A, Rovira T, Viladrich C, et al. Assessing self-reported adherence to HIV therapy by questionnaire: the SERAD (Self-Reported Adherence) Study. AIDS Res Hum Retroviruses. 2007;23(10):1166-75; doi: 10.1089/aid.2006.0120.

54. Vriesendorp R, Cohen A, Kristanto P, Vrijens B, Rakesh P, Anand B, et al. Adherence to HAART therapy measured by electronic monitoring in newly diagnosed HIV patients in Botswana. Eur J Clin Pharmacol. 2007;63(12):1115-21; doi: 10.1007/s00228-007-0369-2.

55. Plipat N, Kottapat U, Komoltri C, Voradilokkul J, Anansakunwatt W, Chearskul P, et al. Evaluation of a practical method to assess antiretroviral adherence in HIV-infected Thai children. Southeast Asian J Trop Med Public Health. 2007;38(5):828-34.

56. Vaz MJ, Barros SM, Palacios R, Senise JF, Lunardi L, Amed AM, et al. HIV-infected pregnant women have greater adherence with antiretroviral drugs than non-pregnant women. Int J STD AIDS. 2007;18(1):28-32; doi: 10.1258/095646207779949808.

57. Holzemer WL, Bakken S, Portillo CJ, Grimes R, Welch J, Wantland D, et al. Testing a nurse-tailored HIV medication adherence intervention. Nurs Res. 2006;55(3):189-97; doi: 10.1097/00006199-200605000-00005.

58. Llabre MM, Weaver KE, Durán RE, Antoni MH, McPherson-Baker S, Schneiderman N. A measurement model of medication adherence to highly active antiretroviral therapy and its relation to viral load in HIV-positive adults. AIDS Patient Care STDS. 2006;20(10):701-11; doi: 10.1089/apc.2006.20.701.

59. Giordano TP, Guzman D, Clark R, Charlebois ED, Bangsberg DR. Measuring adherence to antiretroviral therapy in a diverse population using a visual analogue scale. HIV Clin Trials. 2004;5(2):74-9; doi: 10.1310/jfxh-g3x2-eym6-d6ug.

60. Wohl DA, Stephenson BL, Golin CE, Kiziah CN, Rosen D, Ngo B, et al. Adherence to directly observed antiretroviral therapy among human immunodeficiency virus-infected prison inmates. Clin Infect Dis. 2003;36(12):1572-6; doi: 10.1086/375076.

61. Walsh JC, Mandalia S, Gazzard BG. Responses to a 1 month self-report on adherence to antiretroviral therapy are consistent with electronic data and virological treatment outcome. Aids. 2002;16(2):269-77; doi: 10.1097/00002030-200201250-00017.

62. Murphy DA, Greenwell L, Hoffman D. Factors associated with antiretroviral adherence among HIV-infected women with children. Women Health. 2002;36(1):97-111; doi: 10.1300/J013v36n01_07.

63. Arnsten JH, Demas PA, Farzadegan H, Grant RW, Gourevitch MN, Chang CJ, et al. Antiretroviral therapy adherence and viral suppression in HIV-infected drug users: comparison of self-report and electronic monitoring. Clin Infect Dis. 2001;33(8):1417-23; doi: 10.1086/323201.

64. Frick PA, Gal P, Lane TW, Sewell PC. Antiretroviral medication compliance in patients with AIDS. AIDS Patient Care STDS. 1998;12(6):463-70; doi: 10.1089/apc.1998.12.463.

65. Wall TL, Sorensen JL, Batki SL, Delucchi KL, London JA, Chesney MA. Adherence to zidovudine (AZT) among HIV-infected methadone patients: A pilot study of supervised therapy and dispensing compared to usual care. Drug and Alcohol Dependence. 1995;37(3):261-9; doi: <https://dx.doi.org/10.1016/0376-8716%2894%2901080-5>.
