# Supplementary material for: Discrepancies between self-reported medication in adherence and indirect measurement adherence among patients undergoing antiretroviral therapy: a systematic review
Source: Infect Dis Poverty. 2024 Jul 5;13:51. doi: 10.1186/s40249-024-01221-4 (PMC11225374; doi:10.1186/s40249-024-01221-4)
Supplement: Supplementary file 3 — Supplementary Material 3. [file 40249_2024_1221_MOESM3_ESM.docx]

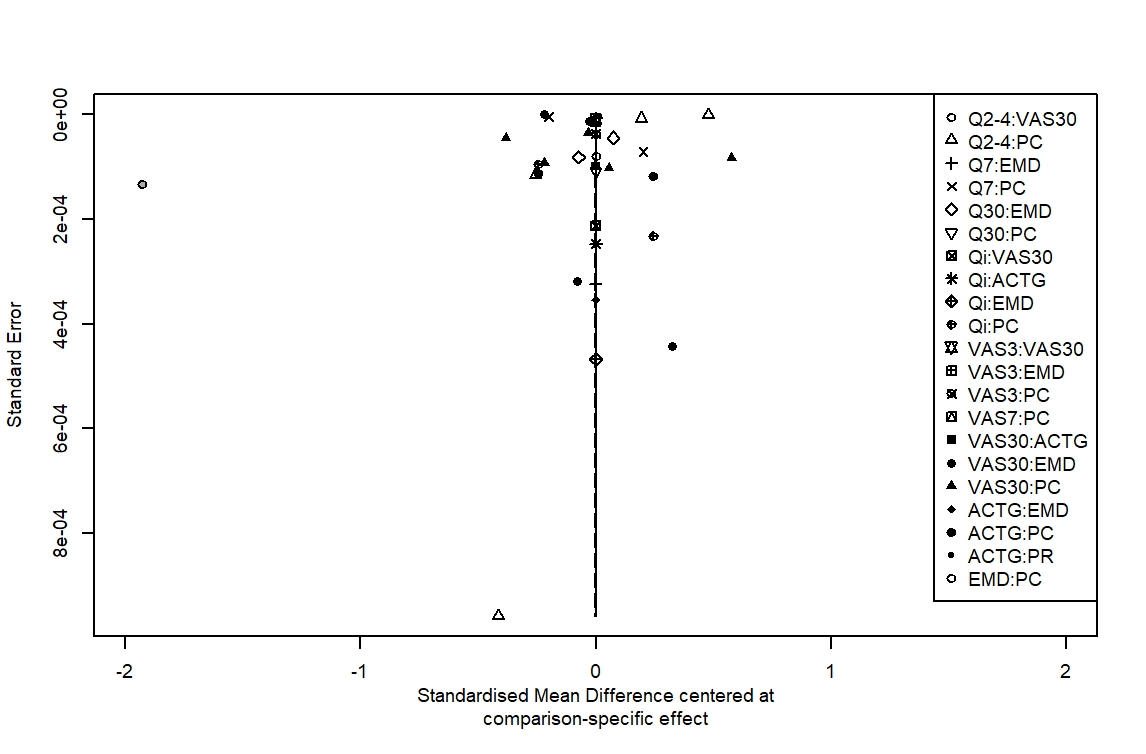


Supplementary Figure 1 Funnel plot for meta-analysis of individual adherence


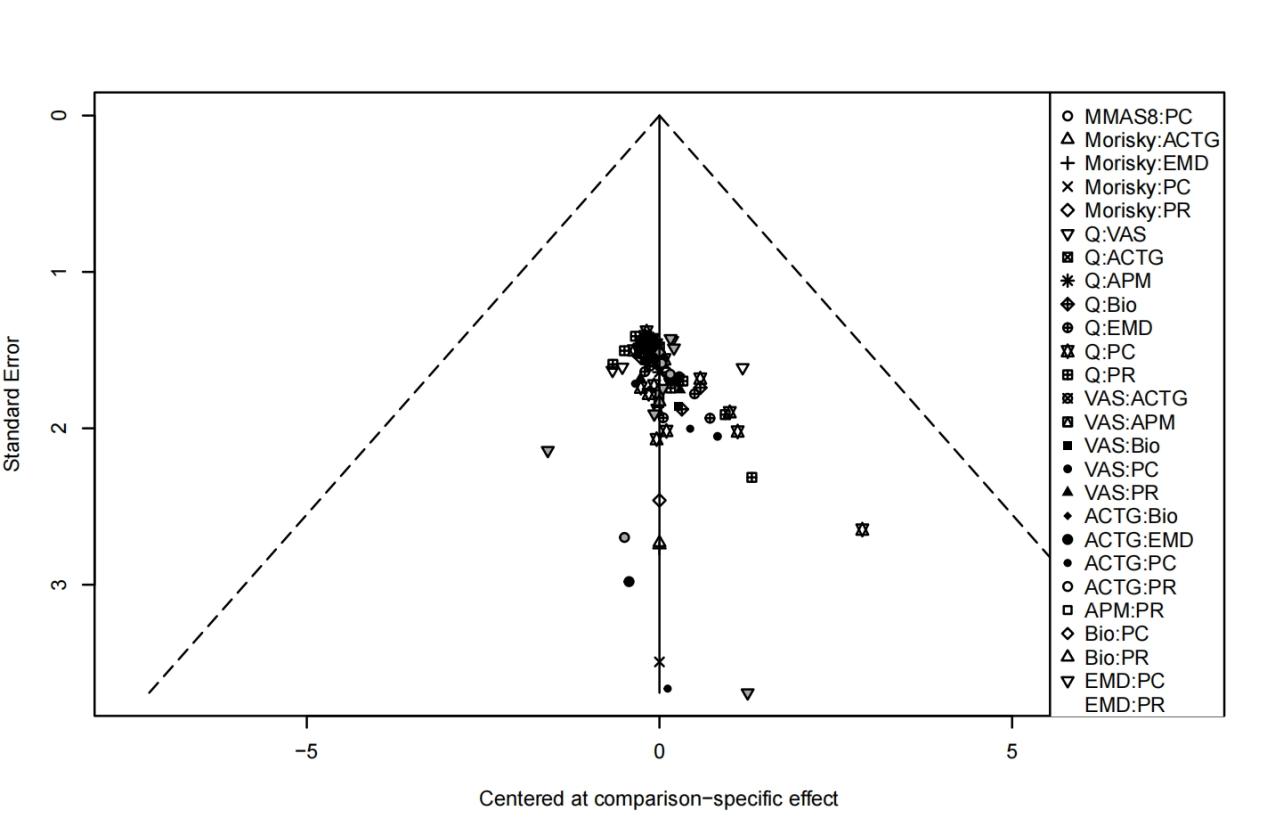


Supplementary Figure 2-1 Funnel plot for meta-analysis of group adherence with questionnaire tools combined


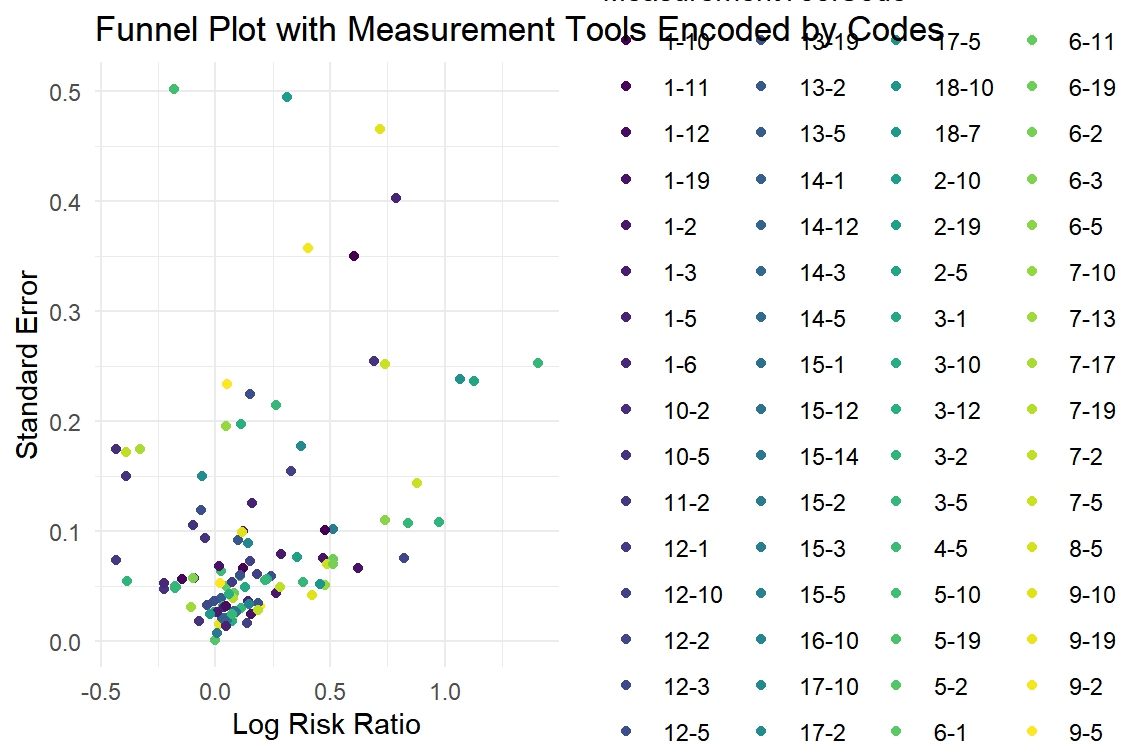


Supplementary Figure 2-2 Funnel plot for meta-analysis of group adherence encoded by codes

Correspondence between Tools and Codes

| Tools | Q30 | PR | Q2-4 | MMAS8 | PC | VAS30 | ACTG | VAS4 | Qnr | Bio |
| --- | --- | --- | --- | --- | --- | --- | --- | --- | --- | --- |
| Code | 1 | 2 | 3 | 4 | 5 | 6 | 7 | 8 | 9 | 19 |
|  |  |  |  |  |  |  |  |  |  |  |
| Tools | EMD | APM | Q7 | VAS | Q90 | Q180 | Q14 | Morisky | Q20 |  |
| Code | 10 | 11 | 12 | 13 | 14 | 15 | 16 | 17 | 18 |  |


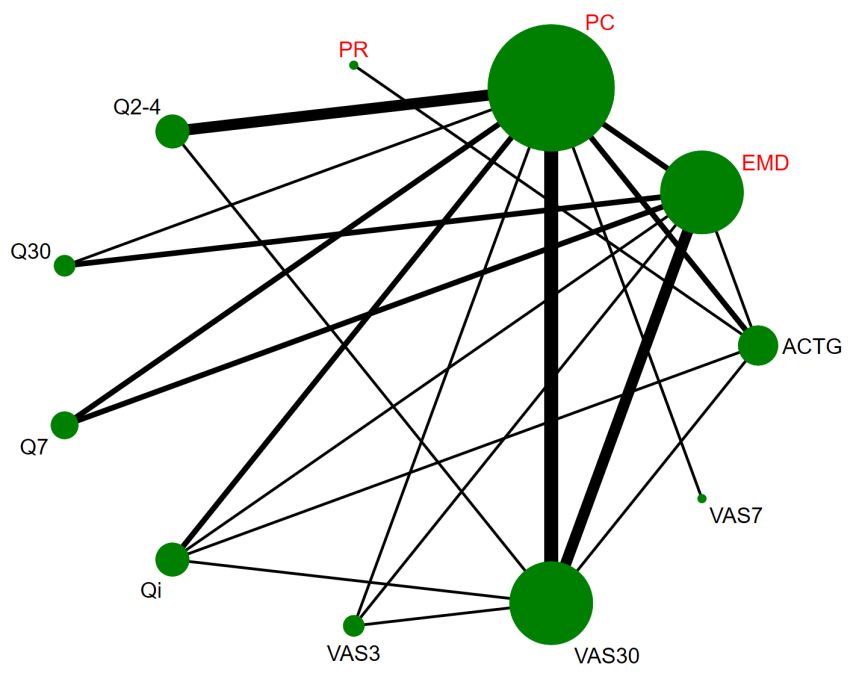


Supplementary Figure 3 Netgraph of of individual adherence measurement tools


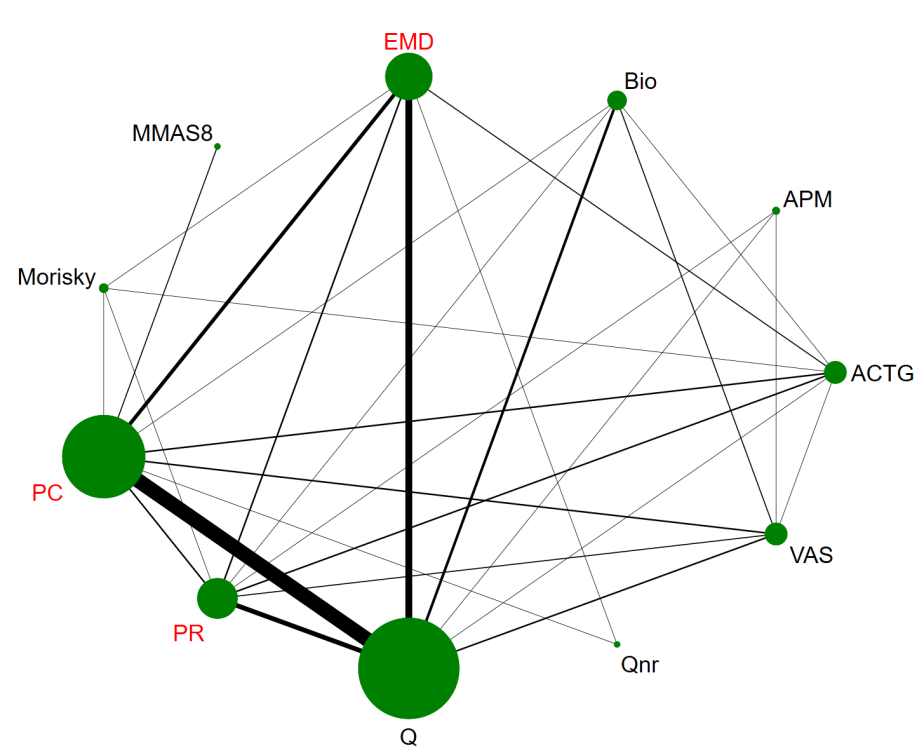


Supplementary Figure 4 Netgraph of group adherence measurement tools

Supplementary Table 1 Sensitivity analysis of individual adherence network mate analysis

A. Exclude the study with the largest effect size

|  | *SMD* | 95% *CI* | *z* | *P* |
| --- | --- | --- | --- | --- |
| ACTG | 0.5225 | (0.3200, 0.7251) | 5.06 | < 0.0001 |
| EMD |  |  |  |  |
| PC | 0.1867 | (0.0335, 0.3399) | 2.39 | 0.0169 |
| PR | 0.3636 | (-0.0687, 0.7959) | 1.65 | 0.0992 |
| Q2-4 | 0.7253 | (0.5029, 0.9478) | 6.39 | < 0.0001 |
| Q30 | 0.3782 | (-0.0037, 0.7601) | 1.94 | 0.0522 |
| Q7 | 0.7023 | (0.4966, 0.9080) | 6.69 | < 0.0001 |
| Qi | 0.1027 | (-0.0998, 0.3053) | 0.99 | 0.3203 |
| VAS3 | 0.6390 | (0.4011, 0.8770) | 5.26 | < 0.0001 |
| VAS30 | 0.4558 | (0.3125, 0.5991) | 6.24 | < 0.0001 |
| VAS7 | 0.635 | (0.2236, 1.0465) | 3.02 | 0.0025 |

*Quantifying heterogeneity: *I^2^* = 100% (95% *CI*: 100.0–100.0%)

B. Exclude small sample studies (*n* ＜ 20)

|  | *SMD* | 95% *CI* | *z* | *P* |
| --- | --- | --- | --- | --- |
| ACTG | 1.0643 | (0.8054, 1.3233) | 8.06 | < 0.001 |
| EMD | . |  |  |  |
| PC | 1.0164 | (0.8352, 1.1976) | 11.00 | < 0.001 |
| PR | 0.9055 | (0.3460, 1.4649) | 3.17 | 0.015 |
| Q2-4 | 1.4906 | (1.2105, 1.7708) | 10.43 | < 1.001 |
| Q30 | -0.6644 | (-0.9570, -0.3718) | -4.45 | < -0.001 |
| Q7 | 1.1171 | (0.8531, 1.3811) | 8.29 | < 0.001 |
| Qi | 0.6445 | (0.3856, 0.9035) | 4.88 | < 0.001 |
| VAS3 | 1.0849 | (0.7776, 1.3921) | 6.92 | < 0.001 |
| VAS30 | 0.9637 | (0.7731, 1.1543) | 9.91 | < 0.001 |
| VAS7 | 1.4647 | (0.9368, 1.9927) | 5.44 | < 0.001 |

*Quantifying heterogeneity: *I^2^* = 100% (95% *CI*: 100.0–100.0%)

Supplementary Table 2 Sensitivity analysis of group adherence network mate analysis

A. Exclude the study with the largest effect size

|  | *RR* | 95% *CI* | *z* | *P* |
| --- | --- | --- | --- | --- |
| ACTG | 1.2460 | (0.0202, 2.4719) | 1.99 | 0.0463 |
| APM | 0.2604 | (-1.7583, 2.2792) | 0.25 | 0.8004 |
| Bio | -0.3963 | (-1.6151, 0.8226) | -0.64 | 0.5240 |
| EMD |  |  |  |  |
| MMAS8 | 0.7305 | (-1.5783, 3.0393) | 0.62 | 0.5352 |
| Morisky | 1.2126 | (-1.5917, 4.0169) | 0.85 | 0.3967 |
| PC | -0.1944 | (-0.9385, 0.5496) | -0.51 | 0.6085 |
| PR | -0.1090 | (-1.0444, 0.8263) | -0.23 | 0.8193 |
| Q | 1.0997 | (0.4256, 1.7737) | 3.20 | 0.0014 |
| VAS | 1.1542 | (-0.0100, 2.3185) | 1.94 | 0.0520 |

*Quantifying heterogeneity : *I^2^* = 0% (95%*CI*: 0%-25.2%)

B. Exclude small sample studies (*n* ＜ 20)

|  | *RR* | 95% *CI* | *z* | *P* |
| --- | --- | --- | --- | --- |
| ACTG | 1.2321 | (-0.0172, 2.4815) | 1.93 | 0.0532 |
| APM | 0.2459 | (-1.7917, 2.2835) | 0.24 | 0.8130 |
| Bio | -0.4113 | (-1.6627, 0.8401) | -0.64 | 0.5194 |
| EMD | . |  | . |  |
| MMAS8 | 0.7078 | (-1.6249, 3.0405) | 0.59 | 0.5521 |
| Morisky | 1.2009 | (-1.6103, 4.0121) | 0.84 | 0.4024 |
| PC | -0.2172 | (-1.0324, 0.5980) | -0.52 | 0.6015 |
| PR | -0.1226 | (-1.0912, 0.8459) | -0.25 | 0.8040 |
| Q | 1.0863 | (-0.3518, 1.8207) | 2.90 | 0.0037 |
| VAS | 1.1381 | (-0.0604, 2.3365) | 1.86 | 0.0627 |

*Quantifying heterogeneity: *I^2^* = 0% (95% *CI*: 0–25.8%)
