# Supplementary material for: Discrepancies between self-reported medication in adherence and indirect measurement adherence among patients undergoing antiretroviral therapy: a systematic review
Source: Infect Dis Poverty. 2024 Jul 5;13:51. doi: 10.1186/s40249-024-01221-4 (PMC11225374; doi:10.1186/s40249-024-01221-4)
Supplement: Supplementary file 4 — Supplementary Material 4. [file 40249_2024_1221_MOESM4_ESM.pdf]

INPLASY - International Platform of Registered Systematic Review and Meta-analysis Protocols

INVOICE REGISTRATION FEES $20.00

PAID

Billing Information
Rujun Liao
22233260@qq.com
West China Hospital of Sichuan University

Status: Authorized

Invoice Number: **2023110040**

Protocol: A systematic review on differences between self-reported medication adherence and indirect measurement adherence in patients with antiretroviral therapy.

Date: 09 November 2023

INPLASY, Inc.

E-mail: editorialmanager@inplasy.com
Address - 600 N Broad St, Suite 5, 3327
Middletown, DE 19709 USA.
Federal EIN - 352706904

INPLASY®
